# An individually randomised controlled multi-centre pragmatic trial with embedded economic and process evaluations of early vocational rehabilitation compared with usual care for stroke survivors: study protocol for the RETurn to work After stroKE (RETAKE) trial

**DOI:** 10.1186/s13063-020-04883-1

**Published:** 2020-12-09

**Authors:** Kathryn A. Radford, Kristelle Craven, Vicki McLellan, Tracey H. Sach, Richard Brindle, Ivana Holloway, Suzanne Hartley, Audrey Bowen, Rory O’Connor, Judith Stevens, Julie Philips, Marion Walker, Jain Holmes, Christopher McKevitt, John Murray, Caroline Watkins, Katie Powers, Angela Shone, Amanda Farrin

**Affiliations:** 1 Division of Rehabilitation and Ageing, School of Medicine, Medical School Queen’s Medical Centre, B-Floor, Nottingham, NG7 2UH UK; 2 Clinical Trials Research Unit (CTRU), Leeds Institute of Clinical Trials Research, University of Leeds, Level 11 Worsley Building, Leeds, LS2 9JT UK; 3 Health Economics Group, Norwich Medical School, University of East Anglia, Room 2.37, Norwich Research Park, Norwich, NR4 7TJ UK; 4 Division of Neuroscience and Experimental Psychology, School of Biological Sciences, University of Manchester MAHSC, Manchester, M13 9PL UK; 5 Academic Department of Rehabilitation Medicine, Leeds Institute of Molecular Medicine, University of Leeds, Level D, Martin Wing, Leeds General Infirmary, Leeds, LS1 3EX UK; 6 Patient and Public Involvement Collaborator, Hampshire, UK; 7 Department of Population Health Sciences, Faculty of Life Sciences & Medicine, King’s College London, 5th Floor Addison House, Guy’s Campus, London, SE1 1UL UK; 8 Different Strokes, Raphael House, Ilford, London, IG1 1YT UK; 9 Lancashire Clinical Trials Unit, School of Health, University of Central Lancashire, Brook Building, Room 217, Preston, PR1 2HE UK; 10 Research and Innovation, Jubilee Conference Centre, Jubilee Campus, Wollaton Road, Nottingham, NG8 1BB UK

**Keywords:** Occupational therapy, Return to work, Vocational rehabilitation, Randomised controlled trial, Internal pilot, Stroke, Protocol, Economic evaluation, Process evaluation

## Abstract

**Background:**

Return to work (RTW) is achieved by less than 50% of stroke survivors. The rising incidence of stroke among younger people, the UK economic forecast, and clinical drivers highlight the need for stroke survivors to receive support with RTW. However, evidence for this type of support is lacking. This randomised controlled trial (RCT) will investigate whether Early Stroke Specialist Vocational Rehabilitation (ESSVR) plus usual care (UC) (i.e. usual NHS rehabilitation) is more clinically and cost-effective for supporting post-stroke RTW, than UC alone.

**Methods:**

Seven hundred sixty stroke survivors and their carers will be recruited from approximately 20 NHS stroke services. A 5:4 allocation ratio will be employed to randomise participants to receive ESSVR plus UC, or UC alone. The individually tailored ESSVR intervention will commence within 12 weeks of stroke onset and be delivered for up to 12 months as necessary by trained RETAKE occupational therapists in the community, participants’ homes or workplaces, and outpatient/inpatient therapy settings, via telephone, email, or SMS text message. Outcome data will be collected via self-report questionnaires administered by post or online at 3, 6, and 12 months follow-up. The primary outcome will be self-reported RTW and job retention at 12 months (minimum 2 h/week). Secondary outcomes will include mood, function, participation, health-related quality of life, confidence, intervention compliance, health and social care resource use, and mortality. An embedded economic evaluation will estimate cost-effectiveness and cost-utility analyses from National Health Service (NHS) and Personal Social Services (PSS) perspectives. An embedded process evaluation will employ a mixed methods approach to explore ESSVR implementation, contextual factors linked to outcome variation, and factors affecting NHS roll-out.

**Discussion:**

This article describes the protocol for a multi-centre RCT evaluating the clinical- and cost-effectiveness of an early vocational rehabilitation intervention aimed at supporting adults to return to work following a stroke. Evidence favouring the ESSVR intervention would support its roll-out in NHS settings.

**Trial registration:**

ISRCTN, ISRCTN12464275. Registered on 26 February 2018.

## Administrative information

**Table:** 

Title {1}	An individually randomised controlled multi-centre pragmatic trial with embedded economic and process evaluations of early vocational rehabilitation compared with usual care for stroke survivors: study protocol for the RETurn to work After stroKE (RETAKE) trial
Trial registration {2a and 2b}.	International Clinical Trials Registry Platform (ISRCTN12464275)
Protocol version {3}	5.0, 18th February 2020
Funding {4}	This study is funded by the National Institute for Health Research Health Technology Assessment (NIHR HTA) programme (ref: 15/130/11)
Author details {5a}	^1^Division of Rehabilitation and Ageing, School of Medicine, B-Floor, Medical School Queen’s Medical Centre, Nottingham NG7 2UH. ^2^Clinical Trials Research Unit (CTRU), Leeds Institute of Clinical Trials Research, Level 11 Worsley Building, University of Leeds, Leeds LS2 9JT. ^3^Health Economics Group, Room 2.37, Norwich Medical School, University of East Anglia, Norwich Research Park, Norwich NR4 7TJ. ^4^Lancashire Clinical Trials Unit, School of Health, Brook Building, Room 217, University of Central Lancashire, Preston PR1 2HE. ^5^ Academic Department of Rehabilitation Medicine, Leeds Institute of Molecular Medicine, University of Leeds, Level D, Martin Wing, Leeds General Infirmary, Leeds LS1 3EX. ^6^Patient and Public Involvement Collaborator, Nottingham, UK. ^7^Different Strokes, Raphael House, Ilford, London IG1 1YT. ^8^Division of Neuroscience and Experimental Psychology, School of Biological Sciences, University of Manchester MAHSC, Manchester M13 9PL. ^9^Department of Population Health Sciences, Faculty of Life Sciences & Medicine, King’s College London, 5th Floor Addison House, Guy’s Campus, London SE1 1UL. ^10^Research and Innovation, Jubilee Conference Centre, Jubilee Campus, Wollaton Road, Nottingham NG8 1BB.
Name and contact information for the trial sponsor {5b}	Ms Angela Shone Head of Research Governance, Financial & Business Services Research and Innovation Jubilee Conference Centre Jubilee Campus, Wollaton Road Nottingham, NG8 1BB Phone: 0115 846 7906 Email: angela.shone@nottingham.ac.uk
Role of sponsor {5c}	The trial sponsor is an individual representing the University of Nottingham. They will provide study oversight, have responsibility for systems being in place to set up, run and report the research project, provide regulatory and ethical advice, and act as the legal signatory. The sponsor will have responsibility for decisions regarding study conduct and contracts for researchers, collaborators and study committee members.

## Introduction

### Background and rationale {6a}

The UK incidence of stroke has increased among younger people, with an estimated 41% occurring among those aged under 69 years [1]. Nearly two thirds of stroke survivors are discharged with a residual disability [2], resulting from physical, cognitive, and/or language impairments [3, 4]. Return to work (RTW) is a major goal for many stroke survivors, but less than 50% of those working at stroke onset return [5], often due to the limitations posed by residual disabilities [3, 4], their beliefs [6], employer attitudes [7], and access to timely rehabilitation [8]. Vocational rehabilitation (VR) is a process that supports those disadvantaged by illness or disability to access, return to, and maintain employment or another useful occupation [9].

Work is essential for supporting health, wellbeing, and longevity [10], and protects against social exclusion by providing an income, social interaction, a core role, identity, and purpose [10, 11]. Conversely, long-term worklessness has been linked to increased risk of depression, suicide, reduced quality of life, cardiovascular disease, and health-harming behaviours [11, 12]. The societal cost of stroke has been estimated at £26 billion per year, including £8.6 billion for health and social care [13]. These costs will likely increase due to strokes occurring at earlier ages, improvements in survival rates, and changes in retirement age provisions [1, 14]. The effects on the UK economy may be further compounded by rises in unemployment resulting from the COVID-19 pandemic [15]. The need for support for stroke survivors with RTW is already recognised in policy and clinical guidelines [16–23] and is considered a UK government priority and National Health Service (NHS) outcome [24–26]. Despite this, there has been little evidence reported for the effectiveness of post-stroke VR interventions. A systematic review (2011) of VR post-stroke [27] identified only one randomised controlled trial (RCT) in the United States (US) (*N* = 22, 7 people with stroke) that used a case-coordination approach, leading to 64% employment compared to 36% in usual care (UC) at 6 months [28]. In a subsequent RCT in South Africa (*N* = 94), a 6-week workplace intervention delivered by an occupational therapist (OT) and physiotherapist led to 60% employment at 6 months compared with 20% in UC [29]. However, both RCTs were conducted in non-UK settings with small sample sizes; an adequately powered RCT is needed to replicate the results in UK NHS settings.

Early Stroke Specialist Vocational Rehabilitation (ESSVR) was developed from VR practice recommendations in acquired brain injury [17] and guidance from stroke specialists, VR experts, and stroke survivors. ESSVR combines conventional OT with case coordination [30] and is intended for delivery in the community as often as required by individuals, as determined by a stroke specialist OT with additional VR training. ESSVR includes the following: (a) assessing stroke impact on the person and their job; (b) educating individuals, employers, and families about stroke impact on work, and strategies to lessen impact (e.g. memory aids, fatigue management); (c) work preparation, including opportunities to practice work skills; and (d) liaison with employers and employment advisors to plan and monitor a phased return to work (RTW).

In a single-centre feasibility RCT, ESSVR was compared to usual NHS rehabilitation (UC) in previously employed stroke survivors [31]. In total, 46 out of 92 eligible participants agreed to participate (36 men, mean = 56 years, SD = 12.7). Among these, 34 (74%) had mild-to-moderate strokes, and 32 (70%) were followed up at 12 months follow-up. The study showed it is possible to recruit from an acute stroke unit, randomise to the trial, deliver ESSVR, and measure its effects and costs at 3, 6, and 12 months post-stroke. In the ESSVR group, twice as many people were in work at 12 months post-stroke than with UC, and those in work were less depressed, suggesting potential health benefits [32]. However, this was a small single-centre feasibility RCT, so it is unknown whether observed differences were related to ESSVR or due to chance.

The rising incidence of stroke among younger adults, the UK economic forecast, and clinical drivers highlight the need for stroke survivors to receive support with RTW. However, lack of evidence for the clinical- and cost-effectiveness of stroke-specialist VR has hindered the development and commissioning of VR services [31]. In 2015, only 27% of Clinical Commissioning Groups (CCGs) in the UK commissioned VR services within acute stroke service providers [33]. An adequately powered multi-centre study is needed to inform the clinical- and cost-effectiveness of ESSVR and UC, alongside a process evaluation (methods to be reported elsewhere) exploring contextual factors affecting clinical implementation and RTW outcomes. Evidence in favour of the ESSVR intervention would support its roll-out within the NHS.

The aim of the RETAKE trial is to evaluate the clinical- and cost-effectiveness of ESSVR (plus UC) versus UC alone for helping people return to work after stroke. The null hypothesis is that there is no difference between ESSVR (plus UC) versus UC alone.

### Objectives {7}

The primary objective of this study is to establish whether ESSVR (plus UC) is more effective than UC alone for improving participants’ self-reported RTW (≥ 2 h per week) at 12 months post-randomisation.

The secondary objectives include the following:

- To investigate whether the intervention leads to improvement in self-reported work outcomes, including (a) RTW with the same employer, (b) number of hours worked per week, and (c) number of days worked.
- To investigate whether the intervention improves mood, physical function, participation, health-related quality of life, work self-efficacy, and post-stroke confidence.
- To determine whether the intervention changes overall health (including accident and emergency visits, inpatient admissions, outpatient visits, and primary care use including medications) and social care resource use.
- To estimate the likely cost-effectiveness of the intervention compared to UC alone, from the perspective of the NHS and Personal Social Services (PSS).
- To establish whether the intervention reduces carer burden.

### Trial design {8}

This trial protocol has been reported in line with the Standard Protocol Items: Recommendations for Interventional Trials (SPIRIT) checklist [34]. RETAKE is a pragmatic, multi-centre, superiority randomised controlled trial (RCT) with two parallel groups, and a partially nested trial design with embedded internal pilot and economic and process evaluations. The internal pilot will include 8 sites and will assess whether progression criteria thresholds are met for recruitment rates after 6 months of recruitment and for follow-up rates after 12 months of recruitment (see the ‘Recruitment {15}’ section for further details).

The intervention arm will receive ESSVR in addition to UC, and the intervention will be delivered by ESSVR-trained OTs. This approach has been taken to demonstrate it is feasible to provide the ESSVR intervention as part of routine care across the wider NHS, if benefit is demonstrated. Participants in the UC only arm will receive UC provided by primary care, secondary care, community, and social services.

## Methods: participants, interventions, and outcomes

### Study setting {9}

Participants will be recruited from approximately 20 NHS stroke services across the UK, including hyper acute stroke units, acute stroke units, stroke rehabilitation units, and linked early supportive discharge and community rehabilitation services. The ESSVR intervention will be delivered one-to-one, face-to-face in the community, in the participant’s home or workplace, in an outpatient therapy setting, or in hospital (when the participant is hospitalised for a long period of time), or via telephone, post, email, or SMS text message.

### Eligibility criteria {10}

#### Participant eligibility

Those meeting all of the following criteria will be eligible to participate in the study:

*Inclusion criteria*

- Age 18 years or over at time of the stroke
- Admitted to hospital with a new stroke (all severities)
- In work at stroke onset (including self-employed, paid, or unpaid)
- Willing and have capacity to provide informed consent to participate in the study
- Sufficient proficiency in English language to contribute to the data collection required

Participants with a language barrier resulting from stroke (e.g. aphasia) will still be considered eligible, as long as capacity to provide consent can be established. Local sites will seek assistance from family members or suitably trained independent clinical professionals, to ensure that individuals satisfying the eligibility criteria can be included wherever possible.

*Exclusion criteria*

- Not intending to work

#### Carer eligibility

A carer will be defined as *a main informal caregiver who provides the participant with support a minimum of once per week*. Carers meeting the following inclusion criteria will be eligible to participate in the study:

*Inclusion criteria*

- Nominated carer of consenting participant
- Willing and have capacity to provide informed consent to participate in the study
- Sufficient proficiency in English language to contribute to the data collection required

#### Site identification and eligibility

Stroke services will be eligible to take part if they meet the following eligibility criteria:

*Inclusion criteria*

- Stroke service able to deliver ESSVR
- Agreement by therapy service managers that a site recruitment target of 2 participants per month is feasible
- Agreement by therapy service managers that ESSVR-trained therapists will not treat participants in the UC only arm

*Exclusion criteria*

- Sites that routinely provide well-defined and active vocational rehabilitation for participants within 12 weeks of stroke

#### Therapist identification and eligibility

OTs experienced in delivering community rehabilitation programmes and/or specialist stroke rehabilitation to stroke survivors will be identified by the site to receive training and to deliver the intervention.

### Who will take informed consent? {26a}

The Principal Investigator (PI) at each site will ensure that anyone responsible for the informed consent process is authorised, trained, and competent according to the ethically approved protocol, principles of Good Clinical Practice (GCP) [35], and Declaration of Helsinki (1996) [36].

Written informed consent will be required prior to the participant undergoing study procedures. The PI or their nominee will explain study details and provide the information sheet (Additional file 1). The PI or nominee will answer questions, ensuring the participant has sufficient time to consider their participation. Where a participant is unable to sign their name or mark the consent form (Additional file 2), they will be asked for verbal consent to participate, as observed by an independent witness such as a staff member, relative, or friend, who will then sign and date the form. Aphasia-friendly versions of participant materials (Additional files 3 and 4) will be utilised where appropriate.

#### Approach and informed consent of carers

During recruitment or at the baseline assessment, consenting participants will be asked if they wish to nominate a carer to participate in the study. Carers will only be approached if they have been nominated, and the participant has verbally consented to them being approached. Nominated carers will be provided with a study information sheet (Additional file 5) (with covering letter and consent to follow-up leaflet if sent to their home address) informing them about the study. Research staff will meet with interested carers to answer any questions, and obtain written informed consent (Additional file 6).

### Additional consent provisions for collection and use of participant data and biological specimens {26b}

Participants will be asked to consent to the secure sharing of identifiable information with the Department for Work and Pensions to request information relating to work status (Additional files 2 and 4).

Additional informed consent will be sought from a randomly selected 5% of participants and their carers (if nominated) from both study arms, and from an additional 5% of ESSVR participants as part of the study’s embedded process evaluation. Further details will be reported in the protocol for the RETAKE process evaluation.

## Interventions

### Explanation for the choice of comparators {6b}

#### Usual care (UC)

The study protocol will not restrict access to UC, in line with the pragmatic trial design [37] and the possibility of heterogeneity for UC treatments for stroke survivors. UC will be available to both intervention and UC (control) participants and will consist of usual NHS rehabilitation provided by primary care, secondary care, community, and social services, as determined by local policies and procedures. It is likely to include General Practitioner (GP) appointments for medical problems, rehabilitation for activities of daily living (e.g. washing and dressing, toileting, cooking, driving/transport use), and use of voluntary sector services.

### Intervention description {11a}

#### ESSVR (plus UC)

ESSVR is an early, individually tailored intervention delivered by stroke specialist OTs who have undergone training delivered by experienced OTs from the central RETAKE team during site set-up. The 2-day face-to-face training package includes provision of an ESSVR manual, clinical scenarios, and case studies to support learning for the delivery of ESSVR. The RETAKE OTs will be briefed via group discussion and case studies on the trial’s design, paperwork to be completed, and issues relating to delivery of the intervention within the trial context. Throughout the study duration, RETAKE OTs will receive a 1-day refresher training day, monthly group mentoring sessions, and ad hoc individualised support via email or telephone.

RETAKE OTs will initiate communication with ESSVR participants via telephone or letter. The content, dose (e.g. number of sessions), intensity, and duration of the intervention will be individually tailored according to participants’ needs, their preferences, and employment context.

RETAKE OTs will follow the ESSVR manual and record delivery of content, duration, and dose on Case Reporting Forms (CRFs) and routine treatment records. Assessments and intervention will include the following:

- Assessing the impact of stroke on the participant and their job
- Educating patients, employers, and families about stroke impact on work
- Finding strategies to lessen impact, e.g. memory aids, pacing to conserve energy
- Work preparation, including establishment of routines and opportunities to practice work skills (e.g. use of computers to increase concentration, walking to increase physical stamina)
- Liaison with employers and employment advisors to plan and monitor a phased RTW (as needed for up to 12 months post-randomisation)

### Criteria for discontinuing or modifying allocated interventions {11b}

#### Withdrawal

Prior to provision of consent, it will be explained that participant withdrawal from the study will not affect their future care or benefits, but any data collected up to that point will be included in analyses and cannot be erased or omitted. Participants may be withdrawn from the study at their own request or at the discretion of the PI or nominee. At the time of withdrawal, it will be clarified whether participants are withdrawing from the intervention and/or data collection.

### Strategies to improve adherence to interventions {11c}

Appointed mentors will assess and monitor RETAKE OTs’ progress, skills, and competency to deliver the intervention, and provide monthly telephone support at individual or group level for up to an hour. RETAKE OTs’ competency will be assessed via case vignettes at the end of training and at 6 months post-randomisation. At 12 months post-randomisation, competency will be assessed through review of case notes for 1 randomly selected participant per OT. Competency scores will indicate whether additional training and mentoring support is required. To account for staff turnover, further site-level training will be available when required.

RETAKE OTs’ adherence to the ESSVR manual and intervention delivery will be monitored via mentoring records, content CRFs, and routine treatment records. A fidelity checklist will be completed during observations in a random 5% of cases. Further details of the study’s process evaluation will be reported elsewhere.

### Relevant concomitant care permitted or prohibited during the trial {11d}

No restrictions will be imposed on UC or any other concomitant care or interventions during the study period.

### Provisions for post-trial care {30}

No arrangements are in place for ancillary or post-trial care beyond routine UC, nor for compensation for non-negligent care occurring from study participation, as per usual practice.

### Outcomes {12}

There is no consensus in the literature about what constitutes a successful work outcome. In this study, the primary outcome will be self-reported return to work of ≥ 2 h per week at 12 months post-randomisation, including pre-stroke or new work roles. It will be measured via a positive response to the question, ‘Are you currently in work (paid or unpaid) for at least 2 h per week?’

Return to education was removed as part of the primary outcome following inspection of data from the feasibility trial [38], where only two participants were full-time students at stroke onset, and following consultation with the Patient and Public Involvement (PPI) group. To reflect this change, the RETAKE trial’s inclusion criteria were amended to increase the age of eligibility from 16 to 18 years, excluding those in full-time education (and not employed) prior to their stroke.

The secondary outcomes will be self-reported at 3, 6, and 12 months post-randomisation, and will include return to with the same employer (yes/no, including self-employed work), mean number of hours worked (to be recorded as a proportion of the pre-stroke working hours), total number of days in work, mood, functional ability, social participation, health-related quality of life, health and social care resource use, work self-efficacy, and confidence.

### Participant timeline {13}

**Fig. 1:**
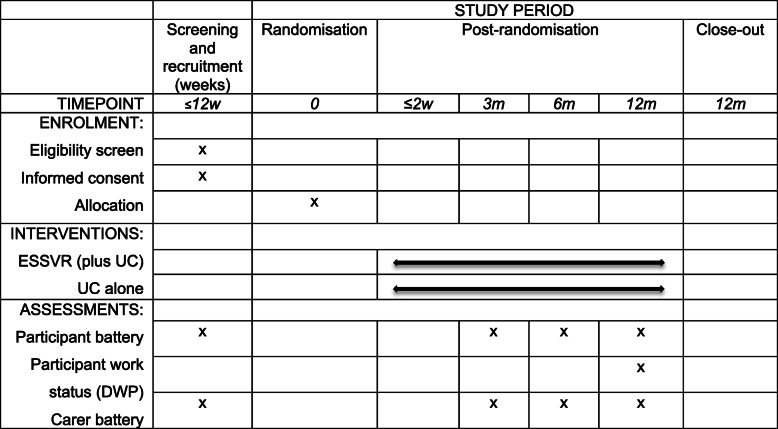
Timeline of screening, recruitment, randomisation, interventions, and assessments

A participant timeline was created according to SPIRIT guidance [34] and is presented in Fig. 1. Following admission into a stroke service, screening, informed consent, and baseline assessments will be completed within 12 weeks of stroke onset, prior to randomisation and allocation. The ESSVR intervention will commence within 2 weeks post-randomisation, and last as long as needed up to 12 months post-randomisation. The start and end dates within the UC control group will likely vary, dependent on the usual NHS rehabilitation provided by each stroke service. Self-reported follow-up questionnaires will be completed by all participants and nominated carers at 3, 6, and 12 months post-randomisation.

### Sample size {14}

The planned study sample size is 760 participants (ESSVR plus UC, 420; UC alone, 340). This provides 90% power at the 5% significance level, to detect a 13% absolute difference in the proportion of people in work at 12 months (assuming 26% in control as per the feasibility study) [32]. It accounts for 20% loss to follow-up and clustering in the intervention arm to account for therapist effects (11 recruited per RETAKE OT, 2 RETAKE OTs per site, 20 sites, intra-cluster correlation coefficient (ICC) 0.03, inflation factor 1.234). Clustering will only exist in the intervention arm to account for between-therapist effects. An ICC of 0.03 has been assumed, given that standardised training and manuals for delivering the intervention will minimise the ICC. A review of similar trials supports an assumption of an ICC no greater than 0.03 [39, 40].

### Recruitment {15}

Recruitment across sites will vary according to service infrastructure and patient pathways. Discussions with sites during site set-up will inform development of tailored strategies to optimise identification and recruitment of participants.

Descriptive data from 8 participating sites will be summarised as part of an internal pilot, to assess whether pre-defined progression criteria thresholds are met at 6 months for recruitment and 12 months for follow-up rates. These assessments will be based on a traffic light system of green (go), amber (review), and red (stop). The recruitment criterion will be a recruitment rate of 2 patients per site per month (green), at least one but less than two (amber), or less than one (red). The follow-up criterion will be a follow-up rate of at least 80% (green), at least 65% but less than 80% (amber), or less than 65% (red). The Trial Steering Committee (TSC) will be provided with the data to inform decisions concerning study continuation. A rescue plan may be developed if significant recruitment and/or follow-up issues are identified.

#### Screening

Following admission to the recruiting unit/service, the patient’s UC team will work closely with experienced and appropriately trained Clinical Research Network (CRN)/local research staff to screen out clearly ineligible patients. The patient’s UC will also provide detailed information about the eligibility criteria for participation in the trial, and will generate a list of potentially eligible participants to track throughout admission and up to 12 weeks post-stroke. Anonymised screening logs will be completed and returned to the Clinical Trials Research Unit (CTRU) regularly to aid identification of recruitment issues.

#### Recruitment in hospital

Recruitment posters with contact details of research staff will be displayed at each site. The UC team will obtain verbal consent from potentially eligible participants to be approached by a researcher. Research staff will approach the patient (and carers if appropriate) and discuss study participation. Those showing interest will be given verbal and written study information, and opportunities to ask questions. They will be asked if they have a carer they wish to nominate, and provided with carer involvement information if appropriate.

The time period given for decisions to participate will vary according to hospital length of stay, and appropriate confirmation of eligibility, written informed consent, and completion of baseline assessments will be completed whilst the patient is still an inpatient.

#### Recruitment post-hospital discharge

Patients expressing interest in the study, who have provided consent to be followed up (by completion of the consent to follow-up leaflet), may be discharged from hospital prior to consent and baseline data collection. In these instances, they will be contacted by research staff to arrange a visit at the patient’s home or hospital to discuss the study further, confirm eligibility, obtain informed consent, and complete the baseline assessments.

In instances where patients were not approached during their hospital stay, their UC team will review their hospital notes for eligibility. Potentially eligible participants will be sent a consent to follow-up leaflet, covering letter, and study information, and asked to complete and return the leaflet within 2 weeks if interested.

Research staff and PIs (or nominees) across inpatient and community settings will be responsible for answering questions, confirming patients’ eligibility, obtaining consent, and performing the baseline assessment.

#### Carer recruitment

Carers will not be approached unless they have been nominated by a participant, and the participant has given verbal consent for them to be approached. Once written informed consent has been obtained from the carer, research staff will arrange to meet them within 2 weeks to perform the baseline assessment.

## Assignment of interventions: allocation

### Sequence generation {16a}

Participants will be individually randomised to ESSVR (plus UC) or UC alone with a 5:4 allocation ratio. The increased proportion allocated to the intervention arm accounts for a greater level of correlation anticipated in the outcomes for those receiving ESSVR, due to participants being treated by the same OT staff. A 24-h computer-generated minimisation programme will be used for allocation, with incorporation of a random element stratified by site, participant age (< 55, ≥ 55), and stroke severity (derived from EQ5D mobility question and Oxford Cognitive Screen (OCS) picture naming and executive tasks).

### Concealment mechanism {16b}

An automated email will be sent from the CTRU 24-h randomisation service to research staff performing the randomisation and the designated RETAKE OTs, confirming randomisation and the participant’s allocation. The site PI will receive an automated email informing them that the participant has been randomised, but no allocation details will be revealed. Participants’ allocation details will only be revealed to PIs following confirmation of the participant’s eligibility, informed consent, and collection of their baseline data.

### Implementation {16c}

The automated email from the CTRU will detail participant identifiers, randomisation allocation, and subsequent actions required. RETAKE OTs will attempt to contact ESSVR participants to inform them of their allocation; they will also keep a record of participants allocated to UC to ensure RETAKE OTs do not treat UC participants.

UC participants will be notified by CTRU of their allocation via letter, with details of subsequent actions (e.g. follow-up assessments). The CTRU will inform participants’ GPs of study participation via letter, omitting allocation details.

## Assignment of interventions: blinding

### Who will be blinded {17a}

Participants and the OTs delivering the intervention will not be blind to allocation group. To minimise the risk of detection bias, baseline data will be collected prior to participant randomisation. Where possible, research staff will be blinded to allocation when they contact participants who have not returned postal/online follow-up questionnaires (following reminder letters/emails).

GPs and the wider health and social care teams will be blinded to participants’ allocation status. ESSVR-directed contact with the GP and wider care teams is permitted as part of the intervention. All reports to the Trial Management Group (TMG) and TSC will be presented in blinded formats.

### Procedure for unblinding if needed {17b}

If requested by the TMG or TSC (e.g. due to safety concerns), CTRU will provide the relevant allocation status. Where unforeseen unblinding occurs, site staff will provide the CTRU with detailed information on circumstances surrounding the incident.

## Data collection and management

### Plans for assessment and collection of outcomes {18a}

The planned timepoints and methods for outcome data collection are summarised in Table 1.

**Table 1:** The planned timepoints and methods for outcome data collection

Assessment	Type	Method of completion	Timeline				
			Screening	Baseline	3 months	6 months	12 months
**Participant data**							
Screening (demographics/assessment of eligibility)	CRF	Researcher	X				
Consent	Consent form	Self-completion	X				
Eligibility/location of baseline assessment	CRF	Researcher		X			
Demographics (age/gender/ethnicity/relationship status/home circumstances/employment details/educational level/driving status)	CRF	Researcher		X			
Oxford Cognitive Screen (OCS)	CRF	Researcher		X			
Details of stroke	CRF	Researcher		X			
Relevant co-morbidities/medical issues	Questionnaire booklet	Researcher/self-completion		X	X	X	X
Contact details (e.g. address/telephone numbers/preferred method of contact/GP details/employer details)	CRF	Researcher		X			
Change of contact details	CRF	Researcher/OT/CTRU		Unscheduled (as made aware)			
Work status	Questionnaire booklet	Researcher/self-completion		X	X	X	X
Hospital Anxiety and Depression Scale (HADS)	Questionnaire booklet	Researcher/self-completion		X	X	X	X
Nottingham Extended Activities of Daily Living (NEADL)	Questionnaire booklet	Researcher/self-completion		X	X	X	X
Community Integration Questionnaire (CIQ)	Questionnaire booklet	Researcher/self-completion		X	X	X	X
Health-related quality of life (EuroQol EQ-5D-5L)	Questionnaire booklet	Researcher/self-completion		X	X	X	X
Resource use (primary care/secondary care/emergency care/medications/social services/wider societal costs (e.g. productivity costs, out of pocket costs))	Questionnaire booklet	Researcher/self-completion		X	X	X	X
Work self-efficacy (single question from the Work Ability Index)	Questionnaire booklet	Researcher/self-completion		X	X	X	X
Confidence after Stroke Measure (CASM)	Questionnaire booklet	Researcher/self-completion		X	X	X	X
Safety reporting	Questionnaire booklet/CRF	Researcher/self-completion			X	X	X
Work status (DWP)	Routine data	Data transfer DWP > CTRU		Downloads to be agreed with DWP to allow 12-month data collection			
Usual care data	CRF	Researcher/self-completion		X	X	X	X
**Carer data**							
Consent	Consent form	Self-completion	X				
Eligibility	CRF	Researcher		X			
Contact details (address/telephone numbers/preferred method of contact/GP details)	CRF	Researcher		X			
Carer demographics (age/gender/ethnicity/relationship to participant/employment details)	Questionnaire booklet	Researcher/self-completion		X	X	X	X
Modified Caregiver Strain Index (MCSI)	Questionnaire booklet	Researcher/self-completion		X	X	X	X
Health-related quality of life (EuroQol EQ-5D-5L)	Questionnaire booklet	Researcher/self-completion		X	X	X	X
Resource use (health, social care, and personal costs)	Questionnaire booklet	Researcher/self-completion		X	X	X	X
Questions relating to impact on carer’s work	Questionnaire booklet	Researcher/self-completion		X	X	X	X

Secondary outcomes not pertaining to work status or days/hours worked will be measured using the following assessment tools:

1. Mood, measured via the Hospital Anxiety and Depression Scale [41] which has been shown to have excellent internal consistency and construct validity among stroke survivors [42].
2. Functional ability, measured via the Nottingham Extended Activities of Daily Living index [43]. This tool measures abilities to carry out instrumental activities of daily living and has established validity among stroke survivors [44].
3. Social participation, measured via the Community Integration Questionnaire [45]. This tool is designed to assess productivity, and home and social integration following acquired brain injury, and has been found to have acceptable internal consistency [46].
4. Health-related quality of life, measured via the EuroQol EQ-5D-5L [47] which records self-ratings of health relating to mobility, self-care, usual activities, pain/discomfort, and anxiety/depression. It has been shown to have construct and convergent validity when tested on a stroke survivor population [48].
5. Health and social care resource use, measured via a bespoke resource use questionnaire.
6. Work self-efficacy, measured via a single question from the Work Ability Index [49], ‘Assume that your work ability at its best has a value of 10 points. How many points would you give your current work ability?’
7. Confidence, measured via the Confidence after Stroke Measure [50]. This tool measures positive attitudes, confidence, and social confidence after stroke, and has established validity and reliability [51].

Research staff will receive training to ensure standardised completion of study-specific assessments. Participants and nominated carers will have the option to complete self-report questionnaires via post or online using internet-based software called QTool.

### Plans to promote participant retention and complete follow-up {18b}

Priming calls, initial and reminder letters/emails, and SMS prompts will be used to maximise data return at all timepoints. A 2-way SMS text message will also be sent (if mobile number provided) to confirm work status only of stroke survivors. If there is no response, the CTRU will alert research staff and, if possible, a blinded staff member will attempt to contact the participant via telephone or arrange a face-to-face visit to perform the assessment.

### Data management {19}

Data collection forms transferred to/from the CTRU will be coded with a study number (made up of the recruitment site code and the participant’s unique sequential trial number), the participant’s initials, and date of birth. Study data will be held securely on paper and electronically at the University of Leeds’ CTRU, and appropriate processes put in place for the transfer, storage, restricted access, and disposal of personal information. Relevant Standard Operating Procedures, Guidelines and Work Instructions in relation to data management, processing, and analysis of data will be followed.

### Confidentiality {27}

All study staff and investigators will endeavour to protect the rights of the trial’s participants to privacy and informed consent. All information collected will be handled strictly in accordance with the consent provided, adhering to the Data Protection Act 2018 [52] at all times. Upon study completion, sites will archive all study data until authorisation for confidential destruction is provided by the study sponsor. Upon study completion, the data shall be transferred from CTRU to the University of Nottingham in an encrypted format and stored for at least 7 years. The Trial Master File and documents held by the CTRU will be archived at secure facilities at the University of Nottingham and University of Leeds.

### Plans for collection, laboratory evaluation, and storage of biological specimens for genetic or molecular analysis in this trial/future use {33}

There are no plans for collection, laboratory evaluation, or storage of biological specimens for this study.

## Statistical methods

### Statistical methods for primary and secondary outcomes {20a}

A detailed statistical analysis plan will be finalised and agreed prior to analysis by the research team. No formal interim analyses are planned. A single final analysis is planned after the trial is closed to recruitment and follow-up and when the full database has been cleaned and locked. Analyses will be completed by the CTRU statisticians using SAS software. Descriptive data (e.g. total numbers of screened patients, patients eligible for participation, those providing consent, reasons for non-entry) will be reported. Baseline characteristics for each study arm will be summarised.

The primary analysis will compare the proportions of participants in work at 12 months post-randomisation between arms, using a partially nested logistic regression mixed-effects model accounting for clustering in the intervention arm only, The model will adjust for stratification factors (site, age, EQ5D mobility score, OCS picture naming score, OCS executive task score) as fixed effects. Therapist (for those in intervention arm) and treatment group will be fitted as random effects. If a participant is unable to report their working status, e.g. if they are dead, this will be taken as a negative response to the question. Other missing data will be assumed missing at random for the primary analysis; multiple imputation for handling such missing data will be explored as a sensitivity analysis.

Analysis of secondary outcomes (return to work with the same employer, number of hours worked per week, number of days in work, mood, physical function, community participation, work self-efficacy, post-stroke confidence) at 3, 6, and 12 months will use a similar modelling strategy to analyse secondary outcome data. Logistic or linear mixed-effects models will be fitted as appropriate.

For all primary and secondary analysis models, corresponding parameter estimates, standard errors, hazard ratios, 95% confidence intervals, *p* values, and ICC (in intervention) will be reported.

### Interim analyses {21b}

No interim analyses are planned.

### Methods for additional analyses (e.g. subgroup analyses) {20b}

Baseline characteristics of those lost to follow-up will be compared with those not lost to follow-up to assess for bias. In addition to the intention to treat (ITT) analysis, Complier Average Causal Effect (CACE) analyses will be undertaken on the primary endpoint in order to measure the impact of the intervention among participants who complied with treatment. Parameter estimates for the intervention effect can then be compared with those from the ITT analysis to help better evaluate the effect of the intervention. Several CACE models will be examined, using increasingly strict definitions of engagement. The CACE models will use the same mixed-effects modelling techniques used in the primary analysis to ensure for reasonable comparisons to be made. Baseline characteristics of those lost to follow-up at the 12-month timepoint will be compared with those not lost to follow-up to assess for bias.

### Methods for economic analysis

A within-trial economic evaluation (a cost-utility analysis) will be conducted comparing the costs and QALYs in the ESSVR plus usual care group to usual care alone group for participants of working age from the perspective of the UK NHS and PSS in the base case as recommended [53] and from a wider perspective in secondary analysis to reflect the expected wider costs and benefits of the VR intervention.

The intervention resource use (comprising training, mentoring, and delivery) will be captured and recorded by the intervention OTs. This will be used to estimate the cost of the training and mentoring components to be used in the main economic evaluation. Levels of wider health, PSS, and societal resource use at baseline, 3, 6, and 12 months will be captured using a bespoke resource use questionnaire designed for self-completion (or with help where required). Although intervention delivery costs will be estimated using data captured by the OTs delivering the interventions, these will not be included in the economic evaluation as it was felt by the research team, which includes PPI members, that participants would not be able to distinguish between intervention OT visits and non-intervention OT visits such that participants are going to be asked explicitly to include all visits in their responses to the resource use questions. To avoid the potential for double counting, we will therefore base resource use and costs on patient reported data only with the exception of training and mentoring costs. We will attach published national unit costs using the common recent price year [54–56] to individual-level quantities of resource use and estimate the mean cost per participant incorporating the cost of the intervention and wider healthcare and PSS resource use (primary care, secondary care, emergency care, medications, and social services). Secondary analysis will take a wider cost perspective including participants, carers, and employers and wider public sector services perspective where possible.

Health-related quality of life will be measured using the EQ-5D-5L [47, 57] at baseline, 3, 6, and 12 months, and valued in line with guidance at the time of analysis [58]. QALYs will be estimated for the trial period using linear interpolation and area under the curve analysis, adjusting for baseline values [59].

A regression-based approach (seemingly unrelated regression equations) [60] will be used for the statistical analysis if the necessary assumptions hold. The level of uncertainty associated with the decision over which option is most cost-effective will be explored using non-parametric bootstrapping [61] to construct the cost-effectiveness acceptability curve (CEAC) [62]. Neither costs nor QALYs will be discounted reflecting the time frame of the trial. Missing data will be handled in line with the approach reported by [63]. Where appropriate, the economic analysis will take the same approach to missing data as the clinical statistical analysis to ensure consistency. Sensitivity analysis will explore the impact of different ways of handling missing data.

The planned economic analysis is subject to change to ensure it stays in line with any changes to accepted methodology during the course of the study. A detailed Health Economics Analysis Plan will be finalised and reviewed by an independent health economist prior to the trial database being locked.

### Methods in analysis to handle protocol non-adherence and any statistical methods to handle missing data {20c}

Analyses will be conducted on all randomised participants, in the study arms to which they were allocated, regardless of intervention non-adherence. Missing data may occur at the item, scale, and timepoint levels. Recommendations on handling of missing item data from scoring protocols will be followed. Otherwise, items will be prorated where 75% or more items for a scale are present. Mechanisms for missing data on variables and key scales will be explored and a multiple imputation model built covering the primary analysis. A sensitivity analysis will explore the impact of employing different missing data handling strategies.

### Plans to give access to the full protocol, participant-level data, and statistical code {31c}

Upon study completion, any party may apply to the Chief Investigator for access to the full protocol, participant-level data, and statistical code for academic research purposes. An information governance committee will govern data access.

## Oversight and monitoring

### Composition of the coordinating centre and trial steering committee {5d}

The TMG will include the Chief Investigator, CTRU, and key external staff members, including two individuals with acquired brain injuries from the PPI group. TMG responsibilities will include clinical set-up of the study, ongoing management, study promotion, and planning for interpretation and dissemination of results. TMG meetings will be held quarterly as a minimum. Further PPI will be provided by the trial’s PPI group, which will include individuals from Different Strokes, Stroke Association, and Headway charities.

The TSC will include an independent chairperson and multiple independent members (including a PPI group member, statistician, and person/s with clinical and trial experience). TSC meetings will take place annually at a minimum to monitor study progress and provide public, clinical, and/or professional advice to TMG members. A TSC sub-committee will review safety issues where necessary.

### Composition of the data monitoring committee, its role and reporting structure {21a}

The CTRU at the University of Leeds will be responsible for monitoring the quality and completeness of study data, in accordance with Standard Operating Procedures (SOPs) and Consolidated Standards of Reporting Trials (CONSORT) guidelines. The TSC will be responsible for reviewing clinical governance issues (in liaison with NHS Trusts, if relevant), and any concerns warranting modification or termination of the study. Decision to terminate the study will be made by the TSC and sponsor.

### Adverse event reporting and harms {22}

Adverse events will be reported if classified as a Related and Unsuspected Serious Adverse Event (RUSAE), i.e. an event that is unexpected in severity and seriousness, and suspected to be related to the study intervention. Possible examples include accidental injury resulting from workplace adaptations recommended by a RETAKE OT, and/or work accidents resulting in injury and hospital treatment.

Self-reported data on adverse events will be collected at 3, 6, and 12 months post-randomisation, or via a site notifying the CTRU or research team. Adverse events will be immediately reported to the Chief Investigator, who will take appropriate medical action, inform and send follow-up information and reports to the REC, and make necessary protocol amendments.

### Frequency and plans for auditing trial conduct {23}

Investigators will notify the CTRU if there is a breach of protocol or GCP principles likely to significantly affect participants’ safety, health, and wellbeing, or scientific value of the research.

### Plans for communicating important protocol amendments to relevant parties (e.g. trial participants, ethical committees) {25}

Important protocol amendments will be made by the Chief Investigator, following consultation with the TMG and any required approval from the REC and Research and Development departments. Amendments will be communicated to participants by the PI, and an amended Informed Consent form completed.

### Dissemination plans {31a}

Study findings will be disseminated, regardless of direction or magnitude of effect, through journal articles, conference presentations, and a peer-reviewed NIHR HTA report submitted within 14 days of study completion. Authorship decisions will be guided by journal criteria. Local collaborators will not have access to study data until publication of the main findings. Participants, study investigators, and PPI group will be informed of results via the study newsletter.

## Discussion

The importance of supporting stroke survivors with RTW is recognised in national policy and clinical guidelines and is a UK government priority and NHS outcome. However, a lack of evidence for the clinical- and cost-effectiveness of stroke-specialist VR has impeded development and commissioning of VR services for this population.

ESSVR is a complex intervention designed to support stroke survivors to access, return to, and maintain working roles in the 12 months following a stroke. A feasibility RCT [31] demonstrated that ESSVR (plus UC) was more effective than UC alone for supporting RTW among stroke survivors, and showed that the intervention could be researched in NHS settings alongside routine care. However, this was a single-centre trial and results were not powered to determine effectiveness. This protocol describes a multi-centre RCT to determine the clinical- and cost-effectiveness of ESSVR plus UC compared with UC alone.

RTW after stroke is considered a key rehabilitation goal and an indicator of recovery [64]. The National Stroke Programme was developed to achieve the aims of the NHS Long Term Plan [25] and highlights that improving access to VR will increase the number of stroke survivors returning to work [65]. Despite this, health services do not always routinely provide VR, consider VR to be within their remit, or address stroke-specific deficits that can impede successful RTW [66]. The need for provision of stroke-specialist VR is becoming increasingly important, as there continue to be rises in the incidence of stroke among younger people, retirement age thresholds, and the societal costs of stroke. This need is further compounded by the impending recession and predicted twofold increase in UK unemployment rates resulting from the COVID-19 pandemic [15].

The National Stroke Strategy [18] and National Institute of Health and Care Excellence (NICE) [19] both recommend that VR be offered to stroke survivors in a timely manner to enable reasonable and necessary adjustments to be made to support their RTW. NICE recommends identifying the demands of the working role and the person’s impairments on work performance, tailoring the VR intervention, educating employers about the Equality Act 2010 and support available, conducting workplace visits, and liaising with employers to establish reasonable accommodations such as phased return or equipment provision [19]. The ESSVR intervention incorporates all of the above recommendations, but also includes consideration of adjustments that can be made to work roles/responsibilities, levels of supervision and support, work preparation activities, and education for stroke survivors, employers/co-workers, and families about stroke-specific disabilities.

The lack of an approved VR pathway within local areas has previously resulted in miscommunication, inadequate training and knowledge of stroke and VR, poor cross-service collaboration, and mild stroke patients receiving little or no support to RTW [66]. The ESSVR intervention is innovative because it encourages collaboration across the workplace and acute and community health services, an approach highly recommended in the aftermath of the COVID-19 pandemic [67]. Occupational therapists delivering the intervention are specially trained to meet the needs of stroke survivors wishing to RTW and encouraged to provide a flexible and responsive service for as long as necessary up to 12 months post-stroke. Individually tailored cross-service interventions of this kind can be more costly in the short-term. However, a pilot study for a similar intervention among people with traumatic brain injuries reported that the mean health costs per person were only £75 greater per annum [68]. In addition, the long-term cost benefits for this intervention could be high. The 6-year follow-up (19 out of 43 participants) for the feasibility RCT demonstrated that most stroke survivors working at 12 months were still working 6 years later (74%; *n* = 14) [37]. People who return to and stay in work contribute more to the economy and are less likely to experience and require expensive treatment for depression [32, 69].

The ESSVR intervention has been designed in accordance with the Medical Research Council’s framework for developing and evaluating complex interventions [70]. This multi-centre RCT includes an embedded economic evaluation to estimate cost-effectiveness and cost-utility analyses from NHS and PSS perspectives. A mixed methods approach will be taken in the process evaluation (reported elsewhere) to identify contextual factors influencing ESSVR implementation, RTW outcomes, and roll-out within NHS stroke rehabilitation services. If the ESSVR intervention was found to be clinically and cost-effective and could be included as part of routine NHS care, it would ensure stroke survivors of working age have a meaningful rehabilitation endpoint (i.e. RTW). RTW will also enable them to develop a sense of purpose, establish routine, improve their self-worth, have greater financial security, increase social contact, and contribute to the economy [71].

There are various practical and operational issues to consider in this study. For example, certain trial processes may require adaptation to suit participants’ individual needs, because stroke survivors can experience problems with fatigue, concentration, attention, communication, vision, and memory [2, 72]. This study includes participants with strokes of varying severities; therefore, research staff will need to be consistently attentive to each participant’s mental capacity and their consent to take part in the study. SMS text reminders and telephone calls will be conducted to support timely completion of self-report follow-up questionnaires, with involvement of carers and/or telephone support where necessary. Potential barriers to intervention delivery and RTW may include lack of participant and/or employer engagement, participants having limited insight into their disabilities, and/or participants’ inability to return to previous working roles. Such issues will be managed on a case-by-case basis through regular discussions between RETAKE OTs and mentors.

Therapists should receive training to equip them for supporting stroke survivors to RTW [66]. In this study, RETAKE OTs will receive a comprehensive 2-day training package including an ESSVR manual, interactive workshops, and refresher days once recruitment has commenced. Tele-mentoring sessions will be provided monthly with additional mentoring support as needed, to support the OTs with clinical reasoning and help to ensure implementation fidelity. Competency assessments at 6 and 12 months into the intervention period will highlight any further training and/or mentoring support needs.

The ESSVR intervention is a bespoke, manualised, early vocational rehabilitation intervention and is not routinely provided within pre-existing VR pathways. To minimise the risk of contamination, a list of recruited participants will be maintained to ensure RETAKE OTs do not treat UC participants referred to their service during the intervention period. Any potential contamination issues will be discussed between RETAKE OTs and mentors and escalated to the Chief Investigator where necessary. Similarly, the RETAKE OTs will be able to receive support from the mentors and central RETAKE team if they experience difficulty—or are unable to deliver the intervention due to personal or practical matters, such as caseload pressures, sickness, and maternity leave. By offering this support, mentors and/or the central RETAKE team will be able to protect the health and wellbeing of the RETAKE OTs and ensure the participant has ongoing ESSVR throughout the intervention period.

The shift from a single-centre to a multi-centre design also means that extra care will be needed to ensure recruitment staff can recruit eligible participants within 12 weeks of their stroke. Prior to set-up, site initiation visits and telephone meetings will be conducted with therapy managers and other relevant staff to ensure the site is able to deliver the intervention. Staffing levels, recruitment targets, and securing of backfill funding for local RETAKE staff time will be included as topics of discussion during such meetings.

The rising incidence of stroke among younger people, the UK’s economic forecast, and clinical drivers highlight the need for stroke survivors to receive support to RTW. This RCT is a component of a programme of work aimed at investigating the implementation of ESSVR among stroke survivors of working age. Evidence for the clinical- and cost-effectiveness of ESSVR would align with political and clinical guidelines and support its roll-out within NHS settings.

## Trial status

Protocol version 5.0, 18 February 2020. Recruitment for the study commenced in May 2018 and was planned for 26 months. Challenges in trial delivery and the impact of the COVID-19 pandemic meant that recruitment was halted. The recruitment completion date is currently under review, but it is estimated that this will take place in December 2020.

## Supplementary Information

**Additional file 1.**

**Additional file 2.**

**Additional file 3.**

**Additional file 4.**

**Additional file 5.**

**Additional file 6.**

**Additional file 7.**

**Additional file 8.**

**Additional file 9.**

## Abbreviations

CACE: Complier Average Causal Effect
CEAC: Cost-effectiveness acceptability curve
CRF: Case Reporting Form
CRN: Clinical Research Network
CTRU: Clinical Trials Research Unit
EQ-5D-5L: EuroQol 5 dimensions and 5 levels
ESSVR: Early Stroke Specialist Vocational Rehabilitation
ITT: Intention to treat
NHS: National Health Service
NICE: National Institute for Health and Care Excellence
NIHR HTA: National Institute for Health Research Health Technology Assessment
OCS: Oxford Cognitive Screen
OT: Occupational therapist
PPI: Patient and Public Involvement
PI: Principal Investigator
PSS: Personal Social Services
QALYs: Quality-adjusted life years
REC: Research Ethics Committee
RCT: Randomised controlled trial
RETAKE: RETurn to work After stroKE
RTW: Return to work
SFQ: Site Feasibility Questionnaire
SOP: Standard Operating Procedure
SPIRIT: Standard Protocol Items: Recommendations for Interventional Trials
TMG: Trial Management Group
TSC: Trial Steering Committee
UC: Usual care
UK: United Kingdom
US: United States
VR: Vocational rehabilitation
