# Supplementary material for: An individually randomised controlled multi-centre pragmatic trial with embedded economic and process evaluations of early vocational rehabilitation compared with usual care for stroke survivors: study protocol for the RETurn to work After stroKE (RETAKE) trial
Source: Trials. 2020 Dec 9;21:1010. doi: 10.1186/s13063-020-04883-1 (PMC7724443; doi:10.1186/s13063-020-04883-1)

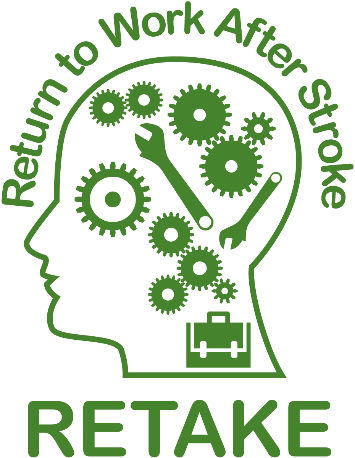


**RETAKE – RET**urn to work **A**fter stro**KE**

# **PARTICIPANT INFORMATION SHEET**

A large-print version of this sheet is available on request.

We are inviting you to take part in a research study called RETAKE. Before you decide if you want to take part, we want to tell you why the research is being done, how we will use the information we get from you, and what the study will involve.

Please read this information carefully, and discuss it with others if you like. Ask us if anything is unclear, or if you would like more information.

**Once you have read this information, the Researcher will talk to you about the study again and you can ask any questions you like.**

- Part 1 tells you why we are doing this study and what will happen if you take part.
- Part 2 gives you more detailed information about the conduct of the study.

**How to contact us**

If you have any questions about this study, please contact:

<<Enter PI, nurse name >>

<< Contact details for site>>

**Thank you for reading this information sheet.**

**Part 1**

**What is the aim of the study?**

Having a stroke can result in problems that affect your chances of returning to work. Some may be visible e.g. mobility problems and some hidden e.g. fatigue or concentration problems. In the early days after stroke it’s not always clear which problems will affect your ability to work.

About a quarter of people who have a stroke are working age but fewer than half return to work. Being in work benefits personal finances, mood, lifestyle and relationships. Current rehabilitation after stroke aims to help people to be able to live independently, but does not always focus on helping them back into work. We have developed a specific way of supporting people who have suffered a stroke, which we hope will help them to return to work. It involves an occupational therapist, experienced in work issues and stroke, working with you, your family and employer to identify whether your stroke might affect you returning to work and if so, to assist you in returning to and remaining in work.

We want to test whether this type of support is more effective than usual stroke care at returning people to work after stroke. This study will compare the two. If findings show the new support is effective it may bring changes to rehabilitation offered to stroke survivors in the future.

We are asking you to consider helping with this study.

**Why have I been chosen?**

Your stroke service is involved in the study. We are inviting all patients who have had a stroke within the last eight weeks, who were employed prior to their stroke to participate. Your care team has helped us to identify who to ask.

**Do I have to take part?**

No. This study is entirely voluntary. If you decide not to take part, you will still receive the same care as those who do take part. If you do agree to take part you are free to withdraw at any time without giving a reason.

If you agree to take part you will be asked to complete a consent form. You will also be asked if you have a person that knows you well (relative/friend/carer) who may like to take part. This is optional and will not affect you taking part.

A research nurse or therapist will then arrange a suitable time with you in the next couple of weeks to meet. This will be in the hospital if you are still an inpatient or at home or in clinic if you have been discharged; whichever is more convenient for you.

**If I want to, will I definitely be able to take part?**

Unfortunately, no. Although we think you might be suitable to take part, we will still need to ask you some questions to make sure you are suitable. This is known as “eligibility screening” and is used to ensure that the study is appropriate for you.

If the eligibility screening shows that it is not appropriate for you to take part we will discuss this with you and will keep a record of why you were not able to take part.

**What will happen to me if I take part?**

You will be asked if you have a person that knows you well (relative/friend/carer) who may like to take part.

This is optional and will not affect you taking part.

1) You will be asked to complete a consent form to confirm you would like to take part in the study.

2) The researcher will collect data from you about you and your stroke, your work, your current health and ability to do everyday activities and any health appointments you attended.

It will take approximately 45 minutes to complete.

3a) Randomly allocated to receive the return to work support (as well as usual stroke care).

4) A therapist will arrange a meeting with you (either at home or at a mutually convenient location) to discuss the return to work support. How often you see the therapist will depend on your needs but may be once every 2-4 weeks for up to 12 months. With your permission, the therapist can meet with you and your employer at work to discuss and carry out any potential return to work plans. You may also continue to receive any usual stroke care alongside the return to work support.

5) You and the person that knows you well (relative/friend/carer if applicable) will be asked to complete questionnaires which will be sent to you at 3, 6 and 12 months after you joined the study (either via post or online). We may also contact you about the study by telephone or text message. You will be asked to complete the questionnaires even if you are not receiving the return to work support - the information you provide is equally important.

If you are contacted by telephone remember the researcher does not know who is receiving the return to work support, so please remember not to talk to them about this.

6) A study researcher may wish to observe some of the support you receive and may ask if you’d like to take part in discussions about your experiences during the study. We would contact you separately about this. You do not have to do this if you do not want to.

You are free to withdraw at any time.

3b) Randomly allocated to continue receiving usual stroke care.

We currently do not know if the return to work support will help stroke survivors get back work so both groups are equally as important for the study.

A relative/friend/carer can help you complete the questionnaire booklets.

You will receive a total of £20 (gift voucher) for completion of the questionnaires as a token of our appreciation.

7) We will look at the therapy records in a sample of people to help us understand what rehabilitation they have received during their time in the study.

## What is randomisation?

Following completion of your questionnaire with the researcher (stage 2 above) you will either be allocated to receive the return to work support plus usual stroke care or to continue receiving usual stroke care only. The best way to compare the two approaches is deciding by chance (‘randomly’) who gets which one - this is called ‘randomisation’. At the end of the study the two groups can be compared to see whether one approach is more helpful than the other. Neither the researchers nor the care team can influence whether you receive the return to work support - this is done randomly by a computer at the Research Office (at the University of Leeds).

## What are the possible disadvantages and risks of taking part?

## We do not expect there are any disadvantages or risks to you. We will arrange any appointments at times to suit you.

## What are the possible benefits of taking part?

This study aims to improve rehabilitation services for people in employment who have had a stroke. We hope that this study will help to support people who have had a stroke in returning to work.

**What happens when the research study stops?**

When the study ends after 12 months, you will continue with whatever usual care is available to stroke survivors via the NHS, your GP or social services in your area. This may or may not include community rehabilitation teams, or stroke association support.

## Will my taking part be kept confidential?

If you decide to participate in the study the information collected about you will be handled strictly in accordance with the consent that you have given and also the 2018 Data Protection Act. Please refer to Part 2 for further details.

**Contact Details**

If you have any questions or would like more information, you can speak to the Researcher, whose details can be found on page 1 of this information sheet.

If you would like further information about research in general, the UK Clinical Research Collaboration (a partnership of organisations working together on research in the UK) have published a booklet entitled ‘Understanding Clinical Trials’. Available here: http://c.ymcdn.com/sites/www.ukcrc-ctu.org.uk/resource/resmgr/2)_understanding_clinical_tr.pdf

**This completes Part 1 of the Information Sheet. If the Information in Part 1 has interested you and you are considering participation, please continue to read the additional information in Part 2 before making any decision**

**Part 2**

**What will happen if I don’t want to carry on with the study?**

Your participation is voluntary and you are free to withdraw at any time, without giving any reason, and without your legal rights being affected. If you withdraw we will no longer collect any information about you or from you but we will keep the information about you that we have already obtained as we are not allowed to tamper with study records and this information may have already been used in some analyses and may still be used in the final study analyses. To safeguard your rights, we will use the minimum personally-identifiable information possible.

**Who has organised, reviewed and funded the research and who will be supervising it?**

This study is organised and run by the University of Nottingham, the Clinical Trials Research Unit (CTRU) at the University of Leeds, Kings’ College London and the University of East Anglia – “the research team”. This study is funded by the Department of Health (National Institute for Health Research) and has been reviewed by East Midlands – Nottingham 2 Research Ethics Committee.

**What if there is a problem?**

In the event that something does go wrong and you are harmed during the research and this is due to someone's negligence then you may have grounds for a legal action for compensation against the University of Nottingham but you may have to pay your legal costs. The normal National Health Service complaints mechanisms will still be available to you.

If you have any worries about this project you should speak to the Researcher or any member of staff. If you remain concerned you can contact your hospital’s Patient Advise and Liaison Service (PALS). Telephone <xxxx xxxxxxx>.

The normal NHS complaints mechanisms will be available to you (if required).

**Will my taking part in this study be kept confidential?**

Under UK Data Protection laws the University of Nottingham and the University of Leeds will act as joint data controllers (legally responsible for the data security) and the Chief Investigator of this study (Dr Kate Radford) is the Data Custodian (manages access to the data). This means that we are responsible for looking after your information and using it properly.

Your hospital will collect information from you and/or your healthcare records for this research study in accordance with our instructions.

Where possible information collected about you for the purposes of this research study (research data), which leaves your hospital will have your name and address removed and a unique code will be used so that you cannot be recognised from it, however sometimes we need to ensure that we can recognise you to link the research data with your healthcare records so in these instances we will need to know your name and date of birth. Your rights to access, change or move your information are limited, as we need to manage your information in specific ways to comply with certain laws and for the research to be reliable and accurate. If you withdraw from the study, we will keep the information about you that we have already obtained and this will still be used in analysing the results of the study.

To safeguard your rights, we will use the minimum personally-identifiable information possible.

The consent form that you sign, which will include your name, will be stored at the CTRU.

Your personal data (e.g. your name, NHS number, address, telephone number(s) and email address (if you have one)) will be shared with the research team. The research team will use this information to contact you about the research study (for example, to send follow-up questionnaires to you online or by post), make sure relevant information about the study is recorded for your care, oversee the quality of the study and to inform you of the outcome of the study and possible follow-up studies (unless you advise us that you do not wish to be contacted).

We may also share personal data (e.g. your name, address, date of birth and National Insurance number) with relevant data providers (e.g. Department for Work and Pensions (DWP)) to request information relating to work status.

At the end of the study your personal data and research data will be securely transferred to the University of Nottingham (sponsor) and a copy stored securely at the CTRU. Personal data and research data will be stored separately to each other and only those who need to will have access to it.

After the end of the study your personal data and research data will be stored securely for 7 years. After this time your data will be disposed of securely. During this time all precautions will be taken by all those involved to maintain your confidentiality, only members of the research team given permission by the data custodian will have access to your data.

Your rights to access, change or move your information are limited as we need to manage your information in specific ways to comply with certain laws and for the research to be reliable and accurate. To safeguard your rights we will use the minimum personally – identifiable information possible.

Most of the information needed for study purposes will be collected on paper forms and sent (usually using standard Royal Mail but in some cases by secure email) to the study team at the CTRU.

Your data will be entered onto secure databases held at the University of Nottingham, CTRU, King’s College London and the University of East Anglia. Only data collected to answer the research question will be used in the study analysis.

Online Questionnaires

If you agree to use the online system (called QTool) to complete your questionnaires, we will email a password to you and provide further details in a User Guide about how to log into the system and change your password. The personal information that we will collect using the QTool System will include email address, password and your questionnaire data. By providing this information, you consent to its collection and use in accordance with the study-specific information that you have been provided.

When your questionnaire data is stored, your personal information will be kept in a secure data centre at the University of Leeds.

**Data Access**

The data collected for the study will be looked at and stored by authorised persons from the research teams at the CTRU, University of Nottingham, King’s College London and University of East Anglia. They may also be looked at by authorised people from regulatory organisations to check that the study is being carried out correctly. All will have a duty of confidentiality to you as a research participant and we will do our best to meet this duty.

Although the information we collect about you is confidential, should you disclose anything to us which we feel puts you or anyone else at risk, we may feel it necessary to report this to the appropriate persons.

You can find out more about how Nottingham use your information and read our privacy notice at: <https://www.nottingham.ac.uk/utilities/privacy.aspx>

**Involvement of the General Practitioner/Family Doctor (GP)**

If you do decide to take part in the study, we will inform your GP and provide them with a copy of this information sheet. We intend to contact your GP to verify your current contact details prior to sending questionnaire booklet. We will use your name, date of birth and NHS number to help with this.

**Future Research**

In accordance with the University of Nottingham’s, the Government’s and our funders’ policies we may share our research data with researchers in other Universities and organisations, including those in other countries, for research in health and social care. Sharing research data is important to allow peer scrutiny, re-use (and therefore avoiding duplication of research) and to understand the bigger picture in particular areas of research. Data sharing in this way is usually anonymised (so that you could not be identified) but if we need to share identifiable information we will seek your consent for this and ensure it is secure. You will be made aware then if the data is to be shared with countries whose data protection laws differ to those of the UK and how we will protect your confidentiality.

**What will happen to the results of the research study?**

Once everyone has finished taking part in the study we will analyse the information we have obtained and will inform you of the outcome. We will also publish study findings in medical papers. You will not be personally identified in any reports or publications.


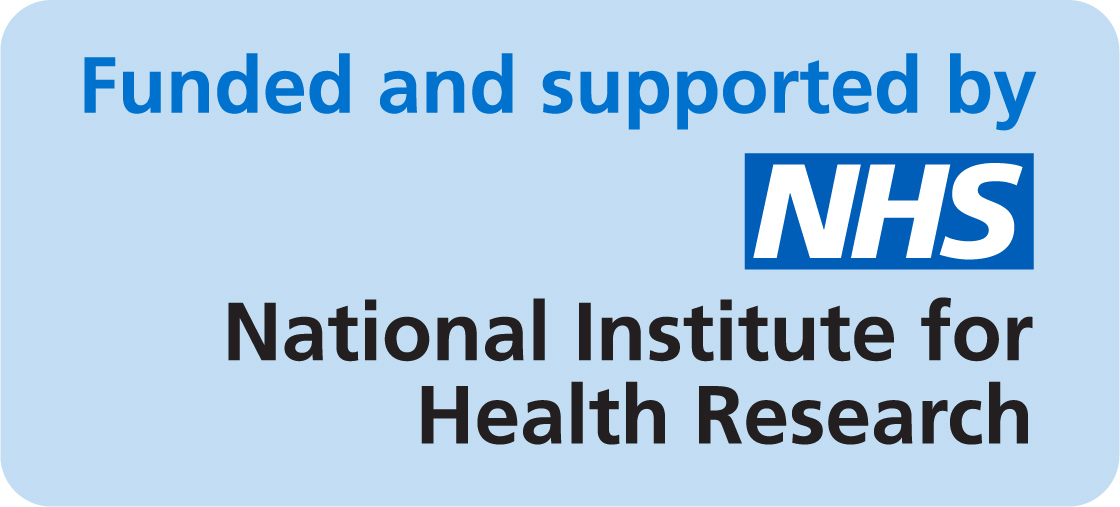

Supplement: Supplementary file 1 — Additional file 1. [file 13063_2020_4883_MOESM1_ESM.doc]
