# Supplementary material for: An individually randomised controlled multi-centre pragmatic trial with embedded economic and process evaluations of early vocational rehabilitation compared with usual care for stroke survivors: study protocol for the RETurn to work After stroKE (RETAKE) trial
Source: Trials. 2020 Dec 9;21:1010. doi: 10.1186/s13063-020-04883-1 (PMC7724443; doi:10.1186/s13063-020-04883-1)

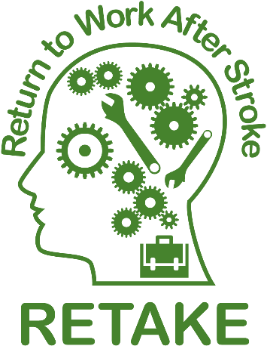


| Participant ID (Site no. / trial no.): | Participant Initials: |
| --- | --- |
| Participant Date of Birth: | Participant NHS/Hospital Number: |
| Principal Investigator: | |

**RETAKE – RET**urn to work **A**fter stro**KE**

**PARTICIPANT CONSENT FORM**

|  | **The following statements are MANDATORY**  You need to agree to all of them to take part in the study | **Please initial each box** |
| --- | --- | --- |
| 1 | I confirm that I have read and understood the Participant Information Sheet dated 18/02/2020 (version 5.0) for the above study. I have had the opportunity to consider the information, ask questions and have had these answered satisfactorily. |  |
| 2 | I understand that my participation is voluntary and that I am free to withdraw at any time without giving any reason, without my medical care or legal rights being affected. |  |
| 3 | I understand that even if I withdraw from the study, the data collected from me up to that point will be used in analysing the results of the study. |  |
| 4 | I understand that relevant section of my healthcare records and data collected during the study may be looked at by authorised individuals from the research team, the University of Nottingham (the study sponsor), the NHS Trust and regulatory authorities where it is relevant to my taking part in this research. I give permission for these individuals to have access to my records. |  |
| 5 | I understand that identifiable information about me may be securely shared with relevant data providers (i.e. the Department for Work and Pensions (DWP)) to request information relating to work status. |  |
|  |  |  |

| 6 | I understand that the information collected about me may be used to support other research in the future, and may be shared anonymously with other researchers. |  |
| --- | --- | --- |
| 7 | As part of this research I understand that a study researcher may wish to observe some of the support I receive and I may be asked if I would like to discuss my experiences of the treatment I have received with a researcher. |  |
| 8 | I understand that my postal address and/or e-mail address and telephone numbers will be passed to the Research Office (at the University of Leeds) for the purpose of completing the questionnaire booklets. |  |
| 9 | I agree for my details and a copy of this consent form (which will include my name and date of birth) to be stored by the Research Office (at the University of Leeds) for the purposes of this study. |  |
| 10 | I agree to my General Practitioner (GP), or any other doctor treating me, being informed of my participation in this study. I agree to a copy of this Consent Form being sent to my GP. |  |
| 11 | I agree to take part in the above study. |  |

| **The following statement is important but OPTIONAL**  Even if you agree to take part in this study, you do not have to agree to this section | | |
| --- | --- | --- |
| 12 | If I am allocated to receive the return to work rehabilitation programme, I give permission for my employer to be contacted. I understand that I will also be asked for verbal consent each time you wish to contact my employer. | **Please tick**  **Yes**  **No** |

**Participant**

Signature:

Name *(block capitals):*

*Day / Month / Year*

………. / ….….... / ………..…

Date:

**Witness (if required)**

Signature:

Name *(block capitals):*

*Day / Month / Year*

………. / ….….... / ………..…

Date:

**Person taking consent**

I have explained the study to the above named participant and he/she has indicated his/her willingness to participate.

Signature:

Name *(block capitals):*

*Day / Month / Year*

………. / ….….... / ………..…

Date:

(1 copy for patient; 1 for the CTRU; 1 held in patient notes, original stored in Investigator Site File)


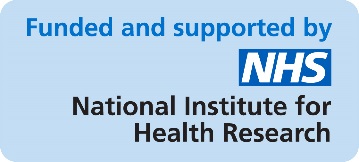

Supplement: Supplementary file 2 — Additional file 2. [file 13063_2020_4883_MOESM2_ESM.docx]
