# Supplementary material for: An individually randomised controlled multi-centre pragmatic trial with embedded economic and process evaluations of early vocational rehabilitation compared with usual care for stroke survivors: study protocol for the RETurn to work After stroKE (RETAKE) trial
Source: Trials. 2020 Dec 9;21:1010. doi: 10.1186/s13063-020-04883-1 (PMC7724443; doi:10.1186/s13063-020-04883-1)

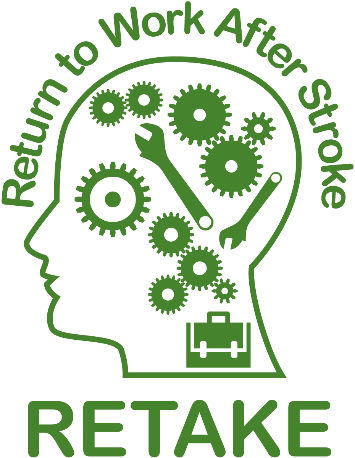


**RETAKE – RET**urn to work **A**fter stro**KE**

# **INFORMATION SHEET**

| **What is the research about?** | |
| --- | --- |
| 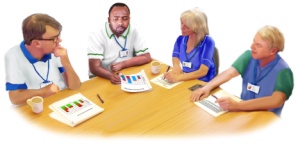 | We are doing some **research**    It is about **returning to work after a stroke**  Research helps us **learn**  We need to **know** more about **how to help people who have had a stroke** |
| **Why are we doing research?** | |
| 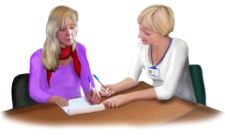 | **Stroke** can make it **difficult to return to work**.  **Current** stroke **care does not always**  **help people get back to work**  after their stroke  We are exploring how best to **help** people **get back to work** after stroke.  An **occupational therapist (OT)** whose job is to **help people return to work** after stroke will help to do this. |
| 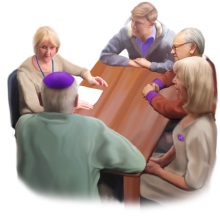 | They will **work with you, your family**  and your **employer** to help you  get back to work.  You will **still receive** the care you would normally get. |
| 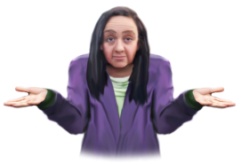 | We **want to know** if this help is **better at helping people get back to work** than the **care they would normally get**. |
| **Who is doing the research?** | |
| 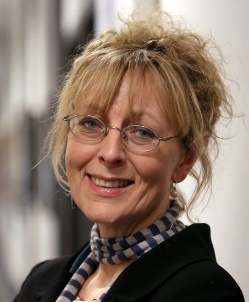 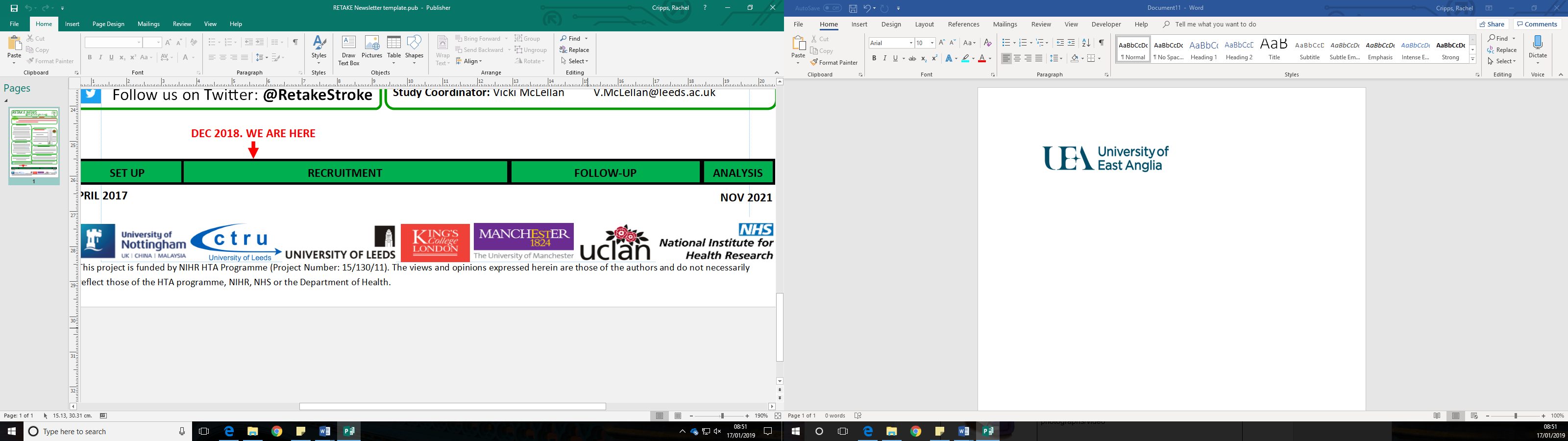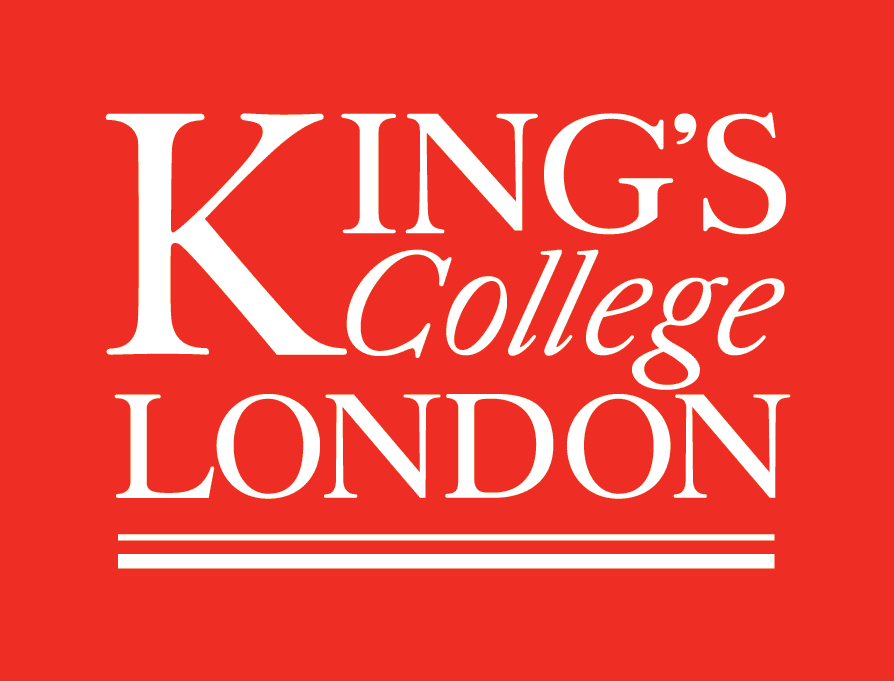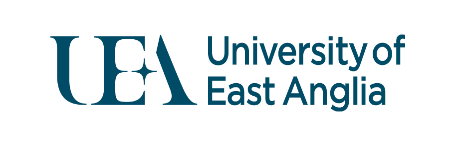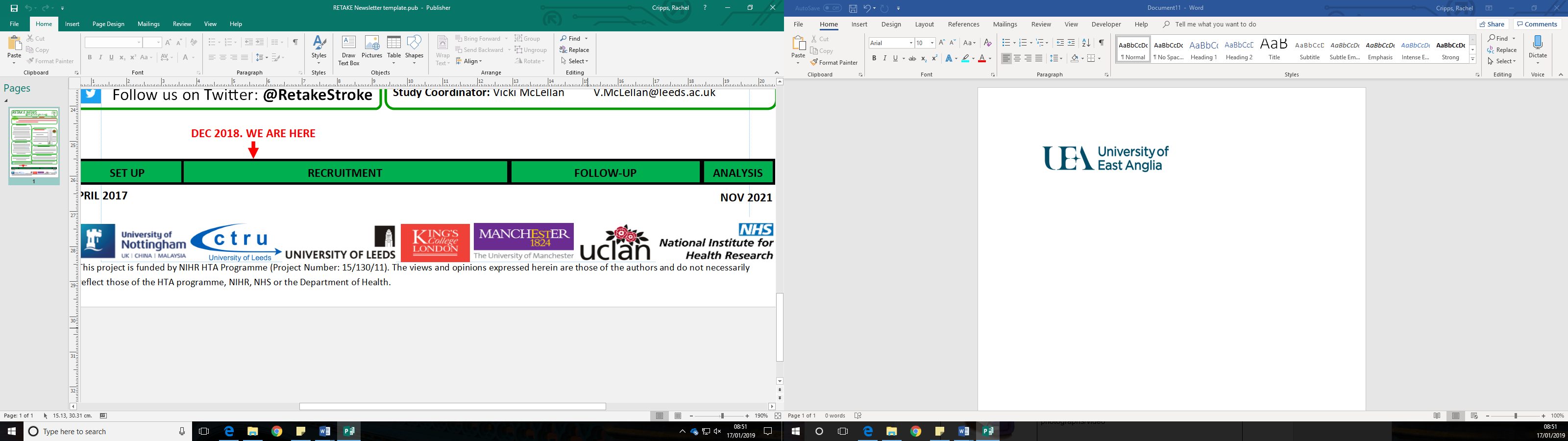 | The **Chief Investigator** is **Dr Kate Radford**  The research is run by the **“research team”** at  **University of Nottingham**  **Clinical Trials Research Unit at the University of Leeds**  **King’s College London**  **University of East Anglia** |
| 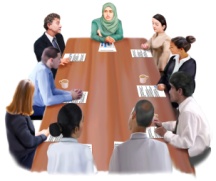 | A committee **decides if research can happen**    This is the **East Midlands – Nottingham 2** **ethics committee**  They say that **this research can happen**  They say that it is **safe**  They say that it has been **planned properly** |
| **Why me?** | |
| 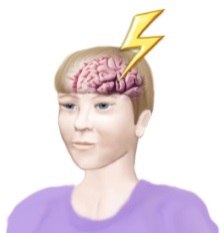 | You have had a **stroke** recently. |
| 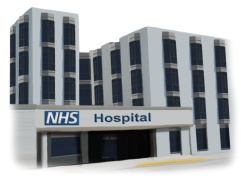 | You **were treated** for your stroke  in a hospital involved with this study. |

**What will I have to do?**

| 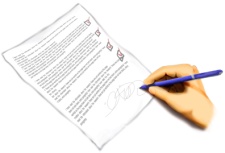 | We would like you to **answer** some **questions** about  **yourself**  and  **Complete some questionnaires**  A **family member, friend**  or the **researcher** can **help** you  These should take around **45 minutes** to complete |
| --- | --- |

| 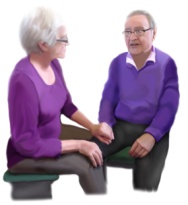 | You will also be asked if you want to  **ask a friend, family member or carer** to **take part**  You can still take part even if your **friend, family member or carer does not.** |
| --- | --- |

| 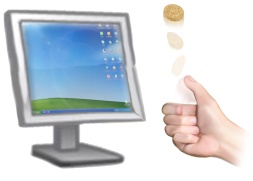 | You will **either** get the **return to work help** or continue to receive the **care you would normally get**  This will be decided by a computer |
| --- | --- |

| **Return to work help** | **Usual stroke care** |
| --- | --- |

| The **OT** whose job is it to help you return to work **will contact you**  You can **choose to see** this person **as much or as little as you want** for a year  If you would like the OT will **talk to your employer** about getting you back to work.  You will **still receive** any other **care you would normally get** |
| --- |

| We will ask you to **complete some questionnaires** on three occasions, **3, 6 and 12 months from now**  We can either send you the **questionnaires by post**  **Or**  You can chose to **complete them on the computer**  You will get a **£20 voucher 12 months from now** for completing your questionnaires |
| --- |

| 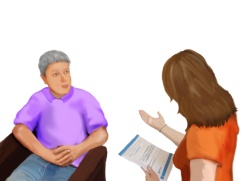The **research team** might **contact you** again to see if they can **watch some of the care you get**  And **ask you some questions** about the **care and services you have had**  You **do not have to do this** if you do not want to |
| --- |

| We will also **look** **at** some people’s **care notes** to see what help they have had because of their stroke. |
| --- |

| **Do I have to take part?** | | | |
| --- | --- | --- | --- |
| 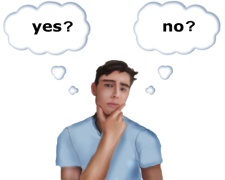 | | | **You can decide**  You **don't have to**  If you don’t take part you will **still get your normal care** |
| 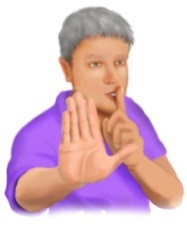 | | | You can **stop** at any time  You **don’t** have to **give a reason**  **If you stop** you will still get your **normal care** |
| 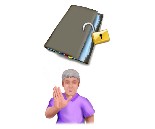 | | | If you stop, we will stop asking you for **information**  We will **keep the information we already have**  It may have already been used in results |
| **Who will see the information about me?** | | | |
| 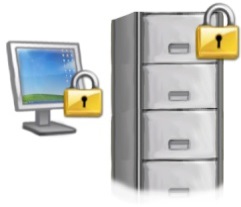 | The **research team** will keep the **information** about you **safe**  The  **research team** will see the **information about you**  **Other people** may need to **look at your information** to check that we are **completing the research properly**  The **research team** will collect your **name, date of birth, address, telephone number, email address (if you have one) and NHS number**  This **will be used** to:   - **Send** you **questionnaires** 3, 6 and 12 months from now - Check **information about you is correct** - Tell you the **results of the research** - Tell you about **other research**   **After the study**, we will **keep your information** **securely** at the University of Nottingham and the Clinical Trials Research Unit in Leeds **for 7 years** | | |
| 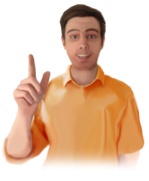 | If the **research team is worried** that you or someone else is **at risk of harm**  we will **tell someone** in your **local healthcare services** | | |
| 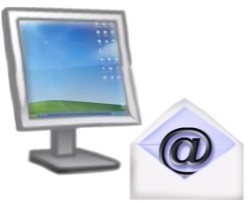 | **Find out more** about how **Nottingham**  **use your information** here:  <https://www.nottingham.ac.uk/utilities/privacy.aspx> | | |
| 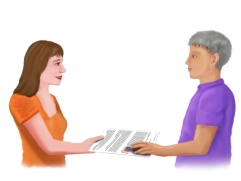 | We may **share your information** (e.g. your name, address, date of birth and National Insurance number) **with** other **people** (e.g. the **Department for Work and Pensions**) to **ask about your work** | | |
| **What might be good about taking part?** | | | |
| 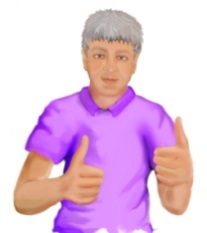 | | | We hope this study will **help people return to work.** |
| **What might be bad about taking part?** | | | |
| 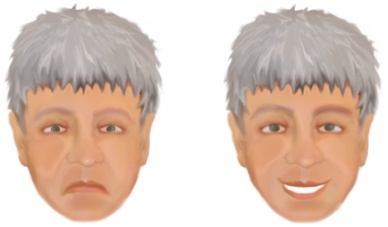 | | | We **don’t think** there will be any **risks**. |
| **Telling your Doctor** | | | |
| 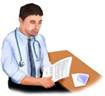 | The **research team** will ask for  **who your doctor’s is** so we can  **contact you again** and **tell them about the research** | | |
| **What will happen after the research?** | | | |
| 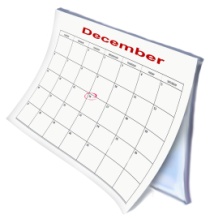 | | The **research** will **end in a year**  **After a year,** you will continue to  **get the care you would normally get** | |
| 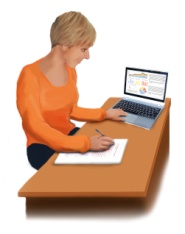 | | The **research teams** will **look at the results** | |
| 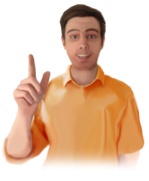 | | They will **learn more** about how best to **help people return to work after stroke** | |
| **What will happen to the results?** | | | |
| 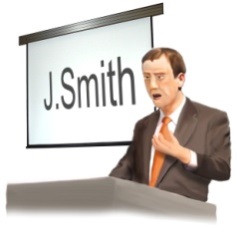 | | We will share the **results** with you and  with **other researchers**  at **conferences** and **meetings**  in **academic journals** | |
| 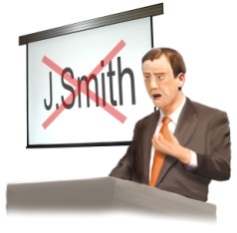 | | The results will **not use your name** | |
| **What next?** | | | |
| 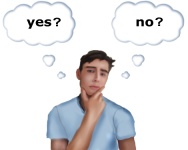 | Do you  **want** to **take part?**  **You need to decide** | | |
| 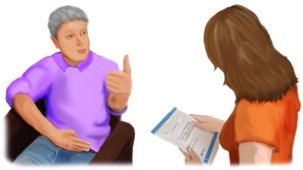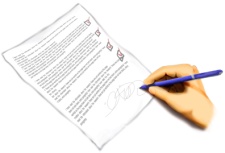 | If you decide to take part  you will need to **sign a consent form**  This says that **you understand** the research and **you agree** to take part | | |
| **What if there is a problem?** | | | |
| 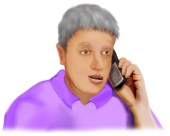  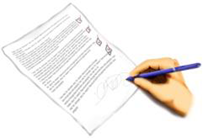 | If  you**take  part**  in  the  research  and  **if  you  think**  you  were  **harmed**  you **may be able to claim compensation** from the **University of Nottingham**  **You** may have to **pay legal costs** | | |
| 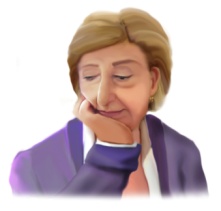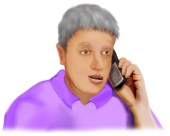 | **If** you have **concerns about the care** you have had in the NHS  or you **need advice** about  **other organisations who can help,**  you can contact:    **The Patient Advice and Liaison Service (PALS)**  on: **[telephone number]** | | |
| **Contact Us** | | | |
| 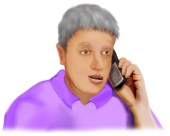 | If you want to **speak to us about the research** please call:  <<Enter PI, nurse name >>  << Contact details for site>> | | |
| 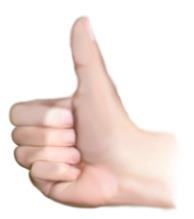  **Thank you** for reading this information sheet | | | |


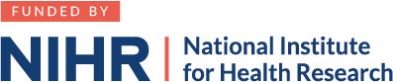

Supplement: Supplementary file 3 — Additional file 3. [file 13063_2020_4883_MOESM3_ESM.docx]
