# Supplementary material for: An individually randomised controlled multi-centre pragmatic trial with embedded economic and process evaluations of early vocational rehabilitation compared with usual care for stroke survivors: study protocol for the RETurn to work After stroKE (RETAKE) trial
Source: Trials. 2020 Dec 9;21:1010. doi: 10.1186/s13063-020-04883-1 (PMC7724443; doi:10.1186/s13063-020-04883-1)

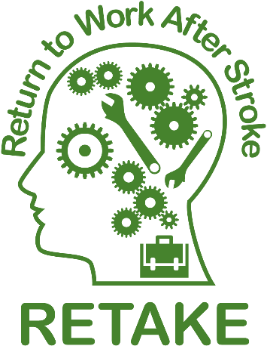


| Participant ID (Site no. / trial no.): | Participant Initials: |
| --- | --- |
| Participant Date of Birth: | Participant NHS/Hospital Number: |
| Principal Investigator: | |

**RETAKE – RET**urn to work **A**fter stro**KE**

**CONSENT FORM**

| **The following statements are MANDATORY**  You need to agree to all of them to take part in the study | | | | | |  |
| --- | --- | --- | --- | --- | --- | --- |
|  | | | | | | **Please [X] each box** 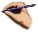 |
| **1** | | 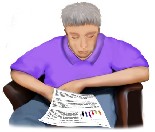I have **read** the **information** about the **research**  (Participant Information Sheet dated 18/02/2020  (version 2.0)) | | | | 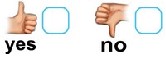 |
|  | | | | | | |
| **2** | | 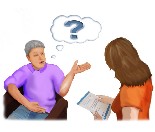I have had the **chance** to **ask questions** about the research.  I have had the opportunity to consider the information, ask questions and have had these answered satisfactorily. | | | | 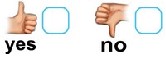 |
|  | | | | | | |
| **3** | | 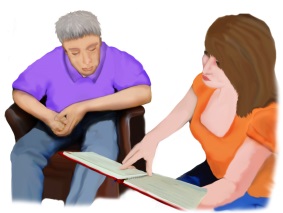I am happy with the **answers** to my questions. | | | | 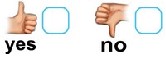 |
|  | | | | | | |
| **4** | | 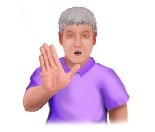  I understand that **I can stop being in the research** at any time. | | | | 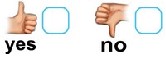 |
|  | | | | | | |
| **5** | | 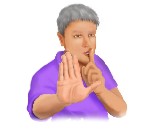  **If I stop, I do not** have to **give a reason** and I will **still get my** **normal care.** | | | | 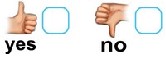 |
|  | | | | | | |
| **6** | | 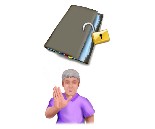  If I stop being in the research, **information already collected** about me will still be used. | | | | 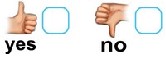 |
|  | | | | | | |
| **7** | | 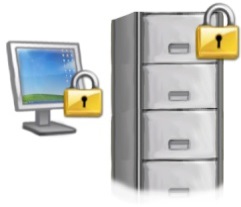I understand that the **research teams** and **other people** may see **information collected about me for this research** to check that the research is being **completed properly**  **I am happy for this to happen** | | | | 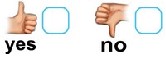 |
|  | | | | | | |
| **8** | | 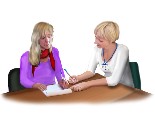I understand that my **information** may be **shared** with other **people** (e.g. the Department for Work and Pensions) to **ask whether or not I am working.** | | | | 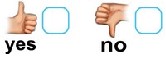 |
|  | | | | | | |
| **9** | | 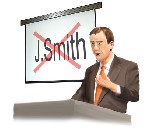 | | I understand that the research team may **share**  **my results** with other  **researchers,** but **not** use my **name** | | 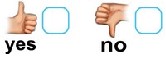 |
|  | | | | | | |
| **10** | | 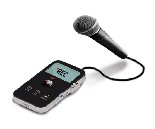 | | I understand that a  researcher may ask to  **watch some of the care I get**  and  **ask me** some questions about the **treatment and services** I have had. | | 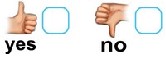 |
|  | | | | | | |
| **11** | | 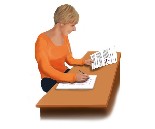 | | I understand that my  **contact information** will be  **sent to the Research Office** in Leeds so I **can be sent** the  **questionnaire booklets** | | 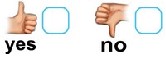 |
| **12** | | 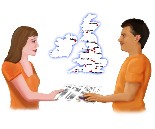 | | I understand that  a **copy** of this **consent form**  (including **my name** and **date of birth**)  will be **stored** by **the Research Office** in Leeds  for study purposes | | 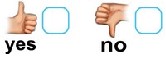 |
|  | | | | | | |
| **13** | | 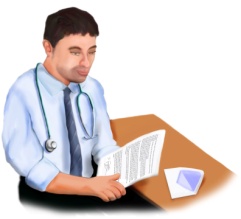 | | I understand that my **doctor**  will be told that  I am **taking part** in  research | | 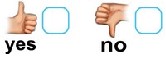 |
|  | | | | | | |
| **14** | |  | | **I agree to take part** in the above study. | | 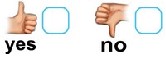 |
|  | |  | |  | |  |
| **The following statement is important but OPTIONAL**  Even if you agree to take part in this study, you do not have to agree to this section | | | | | | |
| **15** |  | | If I get the return to work help I am happy for the therapist to talk to my **employer** if I agree. | | 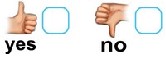 | |

**Participant**

Signature:


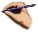


Name *(block capitals):*

Date:

*Day / Month / Year*


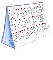
………. / ….….... / ………..…

**Witness (if required)**

Signature:

Name *(block capitals):*

*Day / Month / Year*

………. / ….….... / ………..…

Date:

**Person taking consent**

I have explained the study to the above named participant and he/she has indicated his/her willingness to participate.

Signature:

Name *(block capitals):*

*Day / Month / Year*

………. / ….….... / ………..…

Date:

(1 copy for patient; 1 for the CTRU; 1 held in patient notes, original stored in Investigator Site File)


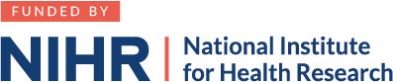

Supplement: Supplementary file 4 — Additional file 4. [file 13063_2020_4883_MOESM4_ESM.docx]
