# Supplementary material for: An individually randomised controlled multi-centre pragmatic trial with embedded economic and process evaluations of early vocational rehabilitation compared with usual care for stroke survivors: study protocol for the RETurn to work After stroKE (RETAKE) trial
Source: Trials. 2020 Dec 9;21:1010. doi: 10.1186/s13063-020-04883-1 (PMC7724443; doi:10.1186/s13063-020-04883-1)

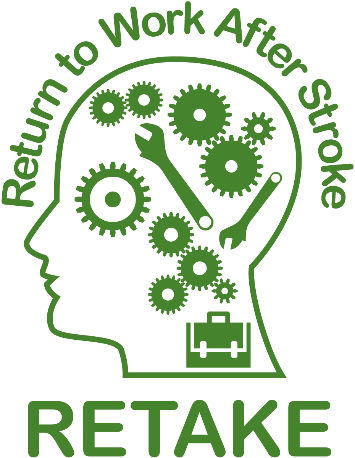


**RETAKE – RET**urn to work **A**fter stro**KE**

# **RELATIVE/FRIEND/CARER INFORMATION SHEET**

A large-print version of this sheet is available on request.

We are inviting you to take part in a research study called RETAKE. Before you decide if you want to take part, we want to tell you why the research is being done, how we will use the information we get from you, and what the study will involve.

Please read this information carefully, and discuss it with others if you like. Ask us if anything is unclear, or if you would like more information.

**Once you have read this information, the Researcher will talk to you about the study again and you can ask any questions you like.**

- Part 1 tells you why we are doing this study and what will happen if you take part.
- Part 2 gives you more detailed information about the conduct of the study.

**How to contact us**

If you have any questions about this study, please contact:

<<Enter PI, nurse name >>

<< Contact details for site>>

**Thank you for reading this information sheet.**

**Part 1**

**What is the aim of the study?**

Having a stroke can result in problems that affect the chance of returning to work. Some may be visible e.g. mobility problems and some hidden e.g. fatigue or concentration problems. In the early days after stroke it’s not always clear which problems will affect the ability to work.

About a quarter of people who have a stroke are working age but fewer than half return to work. Being in work benefits personal finances, mood, lifestyle and relationships. Current rehabilitation after stroke aims to help people to be able to live independently, but does not focus on helping them back into work. We have developed a specific way of supporting people who have suffered a stroke, which we hope will help them return to work. It involves an occupational therapist, experienced in work issues and stroke, working with the stroke survivor, their family and employer to identify whether their stroke might affect them returning to work and if so, assist them in returning to and remaining in work.

We now want to test whether this type of support is more effective than usual stroke care at returning people to work after stroke. This study will compare the two. If findings show the new support is effective it may bring changes to rehabilitation offered to stroke survivors in the future.

We are asking you to consider helping with this study.

**Why have I been chosen?**

Sometimes, when people have a stroke, it can have an effect not just on themselves but also on the health and wellbeing of the people close to them. You have been invited to take

part because you have been identified as being a relative/friend/carer of someone who has had a stroke and they have agreed to take part in this research study.

**Do I have to take part?**

No. This study is entirely voluntary. If you do agree to take part you are free to withdraw at any time without giving a reason.

If you agree to take part you will be asked to complete a consent form.

A research nurse or therapist will then arrange a suitable time with you in the next couple of weeks to meet.

**What will happen to me if I take part?**

1) You will be asked to complete a consent form to confirm you would like to take part in the study.

You are free to withdraw at any time.

2) The researcher will ask you to complete a questionnaire booklet which asks how caring for a stroke survivor could impact on a carer’s wellbeing, work activity and income.

It will take approximately 10 minutes to complete.

The person that you care for will also be asked to complete a questionnaire.

3) You and your relative/friend/the person you care for will be asked to complete questionnaires which will be sent to you at 3, 6 and 12 months after you joined the study (either via post or online). We may also contact you about the study by telephone or text message.

If you are contacted by telephone remember the researcher does not know who is receiving the return to work support, so please remember not to talk to them about this.

4) A study researcher may also ask if you would like to take part in discussions about your experiences during the study. We would contact you separately about this. You do not have to do this if you do not want to.

## What are the possible disadvantages and risks of taking part?

## We do not expect there are any disadvantages or risks to you.

## What are the possible benefits of taking part?

This study aims to improve rehabilitation services for people in employment who have had a stroke. We hope that this study will help to support people who have had a stroke in returning to work and help us to understand the impact of caring for a person who has had a stroke.

## Will my taking part be kept confidential?

If you decide to participate in the study the information collected about you will be handled strictly in accordance with the consent that you have given and also the 2018 Data Protection Act . Please refer to Part 2 for further details.

**Contact Details**

If you have any questions or would like more information, you can speak to the Researcher, whose details can be found on page 1 of this information sheet.

If you would like further information about research in general, the UK Clinical Research Collaboration (a partnership of organisations working together on research in the UK) have published a booklet entitled ‘Understanding Clinical Trials’. Available here: http://c.ymcdn.com/sites/www.ukcrc-ctu.org.uk/resource/resmgr/2)_understanding_clinical_tr.pdf

**This completes Part 1 of the Information Sheet. If the Information in Part 1 has interested you and you are considering participation, please continue to read the additional information in Part 2 before making any decision.**

**Part 2**

**What will happen if I don’t want to carry on with the study?**

Your participation is voluntary and you are free to withdraw at any time, without giving any reason, and without your legal rights being affected. If you withdraw we will no longer collect any information about you or from you but we will keep the information about you that we have already obtained as we are not allowed to tamper with study records and this information may have already been used in some analyses and may still be used in the final study analyses. To safeguard your rights, we will use the minimum personally-identifiable information possible.

**Who has organised, reviewed and funded the research and who will be supervising it?**

This study is organised and run by the University of Nottingham, the Clinical Trial Research Unit (CTRU) at the University of Leeds, Kings’ College London and the University of East Anglia – “the research team”. This study is funded by the Department of Health (National Institute for Health Research) and has been reviewed by East Midlands – Nottingham 2 Research Ethics Committee.

**What if there is a problem?**

In the event that something does go wrong and you are harmed during the research and this is due to someone's negligence then you may have grounds for a legal action for compensation against the University of Nottingham but you may have to pay your legal costs. The normal National Health Service complaints mechanisms will still be available to you.

If you have any worries about this project you should speak to the Researcher or any member of staff. If you remain concerned you can contact your hospital’s Patient Advice and Liaison Service (PALS). Telephone <xxxx xxxxxxx>.

The normal NHS complaints mechanisms will be available to you (if required).

**Will my taking part in this study be kept confidential?**

Under UK Data Protection laws the University of Nottingham and the University of Leeds will act as joint data controllers (legally responsible for the data security) and the Chief Investigator of this study (Dr Kate Radford) is the Data Custodian (manages access to the data). This means that we are responsible for looking after your information and using it properly.

Your hospital will collect information from you for this research study in accordance with our instructions.

Where possible information collected about you for the purposes of this research study (research data), which leaves your hospital will have your name and address removed and a unique code will be used so that you cannot be recognised from it, however sometimes we need to ensure that we can recognise you to link the research data with your healthcare records so in these instances we will need to know your name and date of birth.

The consent form that you sign, which will include your name, will be stored at the CTRU.

Your personal data (e.g. name, address, telephone number(s) and email address (if you have one)) will be shared with the research. The research team will use this information to contact you about the research study (for example, to send follow-up questionnaires to you online or by post), make sure relevant information about the study is recorded for your care, oversee the quality of the study and to inform you of the outcome of the study and possible follow-up studies (unless you advise us that you do not wish to be contacted).

At the end of the study your personal data and research data will be securely transferred to the University of Nottingham (sponsor) and a copy stored securely at the CTRU. Personal data and research data will be stored separately to each other and only those who need to will have access to it.

After the end of the study your personal data and research data will be stored securely for 7 years. After this time your data will be disposed of securely. During this time all precautions will be taken by all those involved to maintain your confidentiality, only members of the research team given permission by the data custodian will have access to your data.

Your rights to access, change or move your information are limited as we need to manage your information in specific ways to comply with certain laws and for the research to be reliable and accurate. To safeguard your rights we will use the minimum personally – identifiable information possible.

Most of the information needed for study purposes will be collected on paper forms and sent (usually using standard Royal Mail post but in some cases by secure email) to the study team at the CTRU.

Every effort will be made to ensure that any further information about you will have your name and address removed so that you cannot be recognised from it.

Your data will be entered onto secure databases held at the University of Nottingham, CTRU, King’s College London and the University of East Anglia. Only data collected to answer the research question will be used in the study analysis.

**Online Questionnaires**

If you agree to use the online system (called QTool) to complete your questionnaires, we will email a password to you and provide further details in a User Guide about how to log into the system and change your password. The personal information that we will collect using the QTool System will include email address, password and your questionnaire data. By providing this information, you consent to its collection and use in accordance with the study-specific information that you have been provided. Further information is provided on the QTool website about how we use cookies and other personal information which is automatically recorded, as with most other web servers.

When your questionnaire data is stored, your personal information will be kept in a secure data centre at the University of Leeds.

**Data Access**

The data collected for the study will be looked at and stored by authorised persons from the research teams at the CTRU, University of Nottingham, King’s College London and University of East Anglia. They may also be looked at by authorised people from regulatory organisations to check that the study is being carried out correctly. All will have a duty of confidentiality to you as a research participant and we will do our best to meet this duty.

Although the information we collect about you is confidential, should you disclose anything to us which we feel puts you or anyone else at risk, we may feel it necessary to report this to the appropriate persons.

You can find out more about how Nottingham and the CTRU use your information and read our privacy notices at: <https://www.nottingham.ac.uk/utilities/privacy.aspx>.

**Involvement of the General Practitioner/Family Doctor (GP)**

We intend to contact your GP to verify your current contact details prior to sending questionnaire booklet. We will use your name and date of birth to help with this.

**Future Research**

In accordance with the University of Nottingham’s, the Government’s and our funders’ policies we may share our research data with researchers in other Universities and organisations, including those in other countries, for research in health and social care. Sharing research data is important to allow peer scrutiny, re-use (and therefore avoiding duplication of research) and to understand the bigger picture in particular areas of research. Data sharing in this way is usually anonymised (so that you could not be identified) but if we need to share identifiable information we will seek your consent for this and ensure it is secure. You will be made aware then if the data is to be shared with countries whose data protection laws differ to those of the UK and how we will protect your confidentiality

**What will happen to the results of the research study?**

Once everyone has finished taking part in the study we will analyse the information we have obtained and will inform you of the outcome by writing to you. We will also publish study findings in medical papers. You will not be personally identified in any reports or publications.


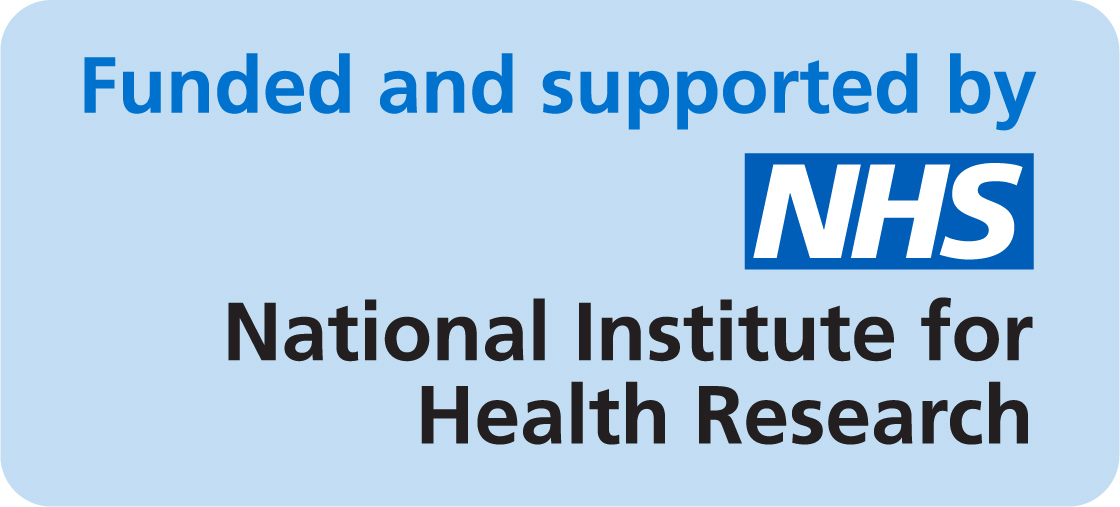

Supplement: Supplementary file 5 — Additional file 5. [file 13063_2020_4883_MOESM5_ESM.doc]
