# Supplementary material for: An individually randomised controlled multi-centre pragmatic trial with embedded economic and process evaluations of early vocational rehabilitation compared with usual care for stroke survivors: study protocol for the RETurn to work After stroKE (RETAKE) trial
Source: Trials. 2020 Dec 9;21:1010. doi: 10.1186/s13063-020-04883-1 (PMC7724443; doi:10.1186/s13063-020-04883-1)

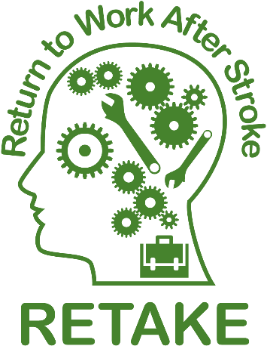


| Participant (Stroke Survivor) ID: |  |
| --- | --- |
| Participant (Stroke Survivor) Initials: | Participant (Stroke Survivor) Date of Birth: |
| NHS/Hospital Number: | Principal Investigator: |

**RETAKE – RET**urn to work **A**fter stro**KE**

**RELATIVE/FRIEND/CARER CONSENT FORM**

|  |  | **Please initial each box** |
| --- | --- | --- |
| 1 | I confirm that I have read and understood the Relative/Friend/Carer Information Sheet dated 07/09/2018 (version 4.0) for the above study. I have had the opportunity to consider the information, ask questions and have had these answered satisfactorily. |  |
| 2 | I understand that my participation in the study is voluntary and that I am free to withdraw at any time without my legal rights being affected. |  |
| 3 | I understand that even if I withdraw from the study, the data collected from me up to that point will be used in analysing the results of the study. |  |
| 4 | I understand that data collected during the study may be looked at by authorised individuals from the research team, the University of Nottingham (the study sponsor), the NHS Trust and regulatory authorities where it is relevant to my taking part in this research. I give permission for these individuals to have access to my records. |  |
| 5 | I understand that the information collected about me may be used to support other research in the future, and may be shared anonymously with other researchers. |  |
| 6 | As part of this research I understand I may be asked if I would like to discuss my experiences during the study with a researcher. |  |
| 7 | I understand that my postal address and/or e-mail address and telephone numbers will be passed to the Research Office (at the University of Leeds) for the purpose of completing the questionnaire booklets. |  |

| 8 | I agree for my details and a copy of this consent form (which will include my name and date of birth) to be stored by the Research Office (at the University of Leeds) for the purposes of this study. |  |
| --- | --- | --- |
| 9 | I agree to my General Practitioner (GP) being informed of my participation in this study, where necessary to verify current contact details. |  |
| 10 | I agree to take part in the above study. |  |

**Relative/Friend/Carer**

Signature:

Name *(block capitals):*

*Day / Month / Year*

………. / ….….... / ………..…

Date:

**Person taking consent**

I have explained the study to the above named relative/friend/carer and he/she has indicated his/her willingness to participate.

Signature:

Name *(block capitals):*

*Day / Month / Year*

………. / ….….... / ………..…

Date:

(1 copy for relative/friend/carer; 1 for the CTRU; original stored in Investigator Site File)


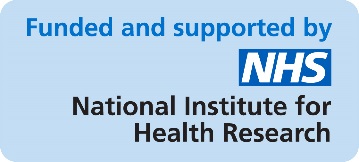

Supplement: Supplementary file 6 — Additional file 6. [file 13063_2020_4883_MOESM6_ESM.docx]
