# Supplementary material for: An individually randomised controlled multi-centre pragmatic trial with embedded economic and process evaluations of early vocational rehabilitation compared with usual care for stroke survivors: study protocol for the RETurn to work After stroKE (RETAKE) trial
Source: Trials. 2020 Dec 9;21:1010. doi: 10.1186/s13063-020-04883-1 (PMC7724443; doi:10.1186/s13063-020-04883-1)
Supplement: Supplementary file 7 — Additional file 7. [file 13063_2020_4883_MOESM7_ESM.pdf]

National Institute for Health Research

Evaluation, Trials and Studies Coordinating Centre
University of Southampton
Alpha House
Enterprise Road
Southampton
SO16 7NS

Tel: 023 8059 5586
Fax: 023 8059 5639
Email: info@netscc.ac.uk
Web: www.netscc.ac.uk

19 January 2017

Dr Kathryn Alice Radford
Associate Professor in Rehabilitation Research
University of Nottingham
Queens Medical Centre
Nottingham
NG7 2UH

Dear Dr Radford

15/130/11 - RETurn to work After stroKE (RETAKE)

Following the HTA Funding Board in November 2016, and the budget review by the HTA Prioritisation Group, I am pleased to inform you that the HTA Programme has recommended funding for your above-titled project, subject to a satisfactory response to the Board's concerns, finance and IP questions detailed below. Please would you inform your co-applicants of this decision.

Please note that this award is not secure until the work has been contracted. Therefore, you must not publicise your achievement until you have received and signed a completed contract from us.

Please address the points raised below in a covering letter alongside an updated version of your full proposal and detailed project description.

Board Feedback

- The applicants should clarify how retention rates will be reported and taken account of during the pilot phase of the study.
- The primary outcome should be more clearly defined and justified. Consideration should be given to whether the threshold of work at 2 hours is appropriate. The applicants should clarify whether and how they will include paid and unpaid work and explain how length of time in work will be dealt with.
- The Board felt that the application was expensive and the costs should be reduced, particularly the costs of statistical support and data management.
- The applicants should explain how contamination will be minimised and monitored.
- The Board were concerned that the heath economics evaluation proposed was restricted to a within study evaluation and the applicants should consider broadening the time period for this.
- The Board noted a large number of discrepancies between the application and the detailed project description and these should be removed in the revised application.

Intellectual Property (IP) Feedback

- We would like to draw your attention to the draft contract, available at http://www.nets.nihr.ac.uk/faqs/finance-and-contracts and specifically to the background IP provisions. Please would you check what third party rights exist in terms of background IP. If there are none, please inform us, as this means there will be no requirement for schedule C in the contract. If third party rights do exist in relation to background IP, please provide details so that they may be appended to the contract in Schedule C.
- It is NIHR's starting position that all arising foreground IP shall be held by the contractor. If you wish NIHR to consider alternative ownership arrangements, please provide details for consideration. The information you provide will feed into Schedule D of the contract.
- Please ensure you submit drafts of any formal agreements that will be put in place, prior to signature, to the HTA. Please advise us as to a potential timeline for these to be submitted.
- The NIHR is very interested in the impact of studies funded and we look forward to seeing detailed dissemination plans in due course.

Finance comments on Research and NHS Costs

Our Finance Department has raised the following queries relating to figures in your application form that you need to respond to:

- Please check the costs for UoL – TBA – Data Manager as they seem incorrect.
- Please confirm that the therapists listed in the staff costs are not actually delivering the intervention.
- Please could you include a more detailed breakdown explaining how the travel costs were calculated.
- Please provide more information on the training delivery expenses.
- Please confirm how many international conferences have been costed. Please note, we will only fund 2 individual attendances at international conferences.
- Please breakdown how the CRF was calculated.
- Please confirm that that no general consumables have been included. We will only fund project specific costs.
- Please provide more information on the randomisation types/registration costs.
- Please provide more information on the following items costed in Other Direct Costs; (1) development of the manual, (2) 3 mentors, (3) Kate Hooban, and (4) Jane Terry.
- Please provide more information and a detailed breakdown of the site payments and per patient payments. Please also outline the activities funded by these costs.
- If ESSVR were to become usual care would travel still be paid for? If so, it is correctly costed, if not it should be a research cost.

If you require any additional advice about the attribution of costs, please contact the Support Costs team, email: netsfin@southampton.ac.uk. Clarification of these costs is essential and they will need to conform to the DH guidance on costs, known as AcoRD, see: https://www.gov.uk/government/publications/guidance-on-attributing-the-costs-of-health-and-social-care-research .

We do not wish to slow down your project start-up but all these points will need to be addressed to the satisfaction of all concerned. The result of any discussions should be outlined in the response letter you send.

The open comments supplied by the peer reviewers concerning your proposal were sent to you to allow you to respond to their comments in advance of the board. These comments are available

under ‘view all my tasks’ in the MIS and they may provide useful expert feedback as you prepare to begin your project.

Involvement of Public Contributors

The HTA Programme recognises the benefit of increasing active involvement of public contributors in research, and recommends that applicants consider whether the scientific quality, feasibility or practicality of their proposal can be improved by involving them. If public contributors are not involved in your current proposal, we encourage you to consider their possible further involvement. Examples of how and why health technology assessment projects have addressed public involvement can be found at www.nets.nihr.ac.uk/ppi

Use of routine data sets

The HTA Programme is interested in taking advantage of the growing utility of routine data (such as HES, GP records etc), and would like investigators, where appropriate, to ask study participants to consent to long term follow up (e.g. beyond the outcomes to be collected in the funded trial) using routinely collected data, and appropriate linkage to allow this data to be best used.

Research Governance

HRA Approval is the process that covers research in the NHS in England. It replaces the need to make separate REC and R&D applications in England. HRA Approval comprises a review by a Research Ethics Committee and an assessment of regulatory compliance and related matters undertaken by dedicated HRA staff. It is being introduced during 2015. Information for applicants is provided on the HRA website at http://www.hra.nhs.uk/resources/hra-approval-applicant-guidance/. Details on the assessment that studies will undergo and the standards that they will be required to meet to receive HRA Approval are available here:
http://www.hra.nhs.uk/resources/hra-approval-applicant-guidance/hra-assessment-criteria-and-standards.

Sponsors and applicants are strongly encouraged to familiarise themselves with the latest version of these standards. Information on the responsibilities of sponsors is provided at http://www.hra.nhs.uk/resources/before-you-apply/roles-and-responsibilties/sponsor.

Integrated Research Application System (IRAS) Identifier

If your project will require ethical approval and/or any other governance approvals, please provide us with the IRAS identifier for this project in your letter of response to the Board, or justification of why it is not required for your project. Please note that you do not need to go through the full process of submitting an ethics request to obtain this; recent changes on the IRAS website http://www.myresearchproject.org.uk/ mean that you are able to obtain an IRAS identifier simply by registering your study. Collecting the identifier at this stage is part of a pilot to try to smooth the permissions and approvals pathway for researchers as part of the Health Research Authority’s work.

Next Steps

Please log into your user account for the NETSCC Management Information System (NETSCC MIS) at https://netscc-mis.nihr.ac.uk to respond to the comments and requirements for changes. You will have access to the relevant parts of your application form to revise and resubmit with the required changes.

Additionally, as part of the submission, you should include a letter that responds to each comment point in turn and clearly explains your proposed action(s). You should also provide a revised copy of your detailed project description which shows all the amendments as ‘tracked changes’.

Both these documents should be included as PDF uploads when submitting your revised application (please note that you should delete any old versions currently showing on the MIS before uploading the revised PDF versions).

Please ensure that we receive your response to the above feedback by **1pm on Thursday 16 February 2017**.

All responses and future communications should be directed to **Roz Bloom, HTA Fund With Change team** at netspostawardsetup@nihr.ac.uk.

Once the contract process is complete, the HTA Programme encourages successful applicants to consider submitting their protocols for review to journals such as the Lancet.

Yours sincerely

Alan Newman
Assistant Research Manager
E-mail: htacet@soton.ac.uk
Direct Tel: 02380 597459

NIHR HTA Programme responsibilities on behalf of the Department of Health

1. To assess the scientific quality of the research as proposed.
2. To establish the value for money of the research proposed.
3. To consider the suitability of the research environment, particularly the experience and expertise of the chief investigator and other key researchers.
4. To ensure the sponsor takes on all appropriate responsibilities before the research begins.
5. To comply and co-operate with any necessary enquiry, audit or investigation.
6. To ensure that the study has ethical and any other necessary approvals before funding is released.
7. To ensure the appropriate indemnities are in place in line with contractual arrangements.
8. To appoint trial management committee chairs and members and their terms of reference.
9. To authorise and approve payments in line with contractual arrangements.
10. To work with the chief investigator to publish information concerning the trial and its findings in the HTA monograph, in line with contractual arrangements.
11. To assess progress, in line with contractual arrangements.
12. To authorise and record changes to protocol, in line with contractual arrangements.
13. To hold site visits and project initiation meetings when appropriate, in line with contractual arrangements.
