# Supplementary material for: An individually randomised controlled multi-centre pragmatic trial with embedded economic and process evaluations of early vocational rehabilitation compared with usual care for stroke survivors: study protocol for the RETurn to work After stroKE (RETAKE) trial
Source: Trials. 2020 Dec 9;21:1010. doi: 10.1186/s13063-020-04883-1 (PMC7724443; doi:10.1186/s13063-020-04883-1)
Supplement: Supplementary file 8 — Additional file 8. [file 13063_2020_4883_MOESM8_ESM.pdf]

East Midlands - Nottingham 2 Research Ethics Committee
The Old Chapel
Royal Standard Place
Nottingham
NG1 6FS

Please note: This is an acknowledgement letter from the REC only and does not allow you to start your study at NHS sites in England until you receive HRA Approval

20 February 2018

Dr Kathryn Radford
Associate Professor in Rehabilitation Research (Long Term Conditions)
University of Nottingham
Division of Rehabilitation and Ageing
B102, Medical School, Queen's Medical Centre (QMC)
Nottingham
NG7 2UH

Dear Dr Radford

**Study title:**	**RETurn to work After stroKE (RETAKE)Early vocational rehabilitation compared with usual care for stroke survivors: an individually randomised controlled multi-centre pragmatic trial with embedded economic and process evaluations**
**REC reference:**	**18/EM/0019**
**Protocol number:**	**17104**
**IRAS project ID:**	**234874**

Thank you for your letter of 6 February 2018. I can confirm the REC has received the documents listed below and that these comply with the approval conditions detailed in our letter dated 05 February 2018.

Documents received

The documents received were as follows:

Document	*Version*	*Date*
Covering letter on headed paper [01_RETAKE REC Cover Letter]	1.0	06 February 2018
Letters of invitation to participant [22_RETAKE Postal Consent to Follow-up Leaflet - Stroke Survivor]	2.0	06 February 2018
Letters of invitation to participant [23_RETAKE Consent to Follow-up Leaflet - Stroke Survivor]	2.0	06 February 2018
Participant information sheet (PIS) [RETAKE Participant Info Sheet_v2.0_TRACKED CHANGES]	2.0	06 February 2018
Participant information sheet (PIS) [RETAKE Participant Info Sheet_v2.0_CLEAN]	2.0	06 February 2018

Approved documents

The final list of approved documentation for the study is therefore as follows:

Document	*Version*	*Date*
Covering letter on headed paper	1.0	15 December 2017
Covering letter on headed paper [01_RETAKE REC Cover Letter]	1.0	15 December 2017
Covering letter on headed paper [01_RETAKE REC Cover Letter]	1.0	06 February 2018
Evidence of Sponsor insurance or indemnity (non NHS Sponsors only) [83_Sponsor Insurance]		
GP/consultant information sheets or letters [46_RETAKE GP Letter]	1.0	15 December 2017
HRA Schedule of Events [80_RETAKE Schedule of Events]	1.0	15 December 2017
HRA Statement of Activities [79_RETAKE Statement of Activities]	1.0	15 December 2017
Interview schedules or topic guides for participants [47_RETAKE Carer Topic Guide - Interviews]	1.0	15 December 2017
Interview schedules or topic guides for participants [48_RETAKE Employer Topic Guide - Interviews]	1.0	15 December 2017
Interview schedules or topic guides for participants [49_RETAKE NHS Staff Topic Guide - Interviews]	1.0	15 December 2017
Interview schedules or topic guides for participants [50_RETAKE Participant Topic Guide - Interviews]	1.0	15 December 2017
Interview schedules or topic guides for participants [51_RETAKE Therapist Topic Guide - Interviews]	1.0	15 December 2017
IRAS Application Form [IRAS_Form_20122017]		20 December 2017
Letter from funder		
Letter from funder [76_Funder Letter]		
Letter from sponsor		
Letter from sponsor [84_Sponsor Letter]		
Letters of invitation to participant [21_RETAKE Postal Approach Cover Letter - Stroke Survivor]	1.0	15 December 2017
Letters of invitation to participant [24_RETAKE Allocation Letter]	1.0	15 December 2017
Letters of invitation to participant [27_RETAKE Participant QTool Follow-up Email]	1.0	15 December 2017
Letters of invitation to participant [33_RETAKE Postal Consent to Follow-up Leaflet - Carer]	1.0	15 December 2017
Letters of invitation to participant [37_RETAKE Carer QTool	1.0	15 December 2017

Follow-up Email]		
Letters of invitation to participant [38_RETAKE Carer QTool Follow-up Reminder Email]	1.0	15 December 2017
Letters of invitation to participant [41_RETAKE Carer Cover Letter - Interviews]	1.0	15 December 2017
Letters of invitation to participant [43_RETAKE NHS Staff Cover Letter - Interviews]	1.0	15 December 2017
Letters of invitation to participant [45_RETAKE Participant Cover Letter - Interviews]	1.0	15 December 2017
Letters of invitation to participant [42_RETAKE Employer Cover Letter - Interviews]	1.0	15 December 2017
Letters of invitation to participant [39_RETAKE Carer Thank You Letter (3/6m)]	1.0	15 December 2017
Letters of invitation to participant [40_RETAKE Carer Thank You Letter (12m)]	1.0	15 December 2017
Letters of invitation to participant [34_RETAKE Consent to Follow-up Leaflet - Carer]	1.0	15 December 2017
Letters of invitation to participant [35_RETAKE Carer Follow-up Letter]	1.0	15 December 2017
Letters of invitation to participant [36_RETAKE Carer Follow-up Reminder Letter]	1.0	15 December 2017
Letters of invitation to participant [28_RETAKE Participant QTool Follow-up Reminder Email]	1.0	15 December 2017
Letters of invitation to participant [29_RETAKE Participant Thank You Letter (3/6m)]	1.0	15 December 2017
Letters of invitation to participant [30_RETAKE Participant Thank You Letter (12m)]	1.0	15 December 2017
Letters of invitation to participant [31_RETAKE QTool Thank You Message]	1.0	15 December 2017
Letters of invitation to participant [32_RETAKE Carer Postal Approach Cover Letter]	1.0	15 December 2017
Letters of invitation to participant [RETAKE Participant Follow-up Letter]	1.0	15 December 2017
Letters of invitation to participant [26_RETAKE Participant Follow-up Reminder Letter]	1.0	15 December 2017
Letters of invitation to participant [44_RETAKE Participant Cover Letter - Interviews]	1.0	15 December 2017
Letters of invitation to participant [22_RETAKE Postal Consent to Follow-up Leaflet - Stroke Survivor]	2.0	06 February 2018
Letters of invitation to participant [23_RETAKE Consent to Follow-up Leaflet - Stroke Survivor]	2.0	06 February 2018
Non-validated questionnaire [60_RETAKE 3m Participant Health Economics Questionnaire]	1.0	15 December 2017
Non-validated questionnaire [61_RETAKE 6m Participant Health Economics Questionnaire]	1.0	15 December 2017
Non-validated questionnaire [62_RETAKE 12m Participant Health Economics Questionnaire]	1.0	15 December 2017
Non-validated questionnaire [63_RETAKE Baseline Carer Health Economics Questionnaire]	1.0	15 December 2017
Non-validated questionnaire [64_RETAKE 3m Carer Health Economics Questionnaire]	1.0	15 December 2017
Non-validated questionnaire [65_RETAKE 6m Carer Health	1.0	15 December 2017

Economics Questionnaire]		
Non-validated questionnaire [66_RETAKE 12m Carer Health Economics Questionnaire]	1.0	15 December 2017
Non-validated questionnaire [59_RETAKE Baseline Participant Health Economics Questionnaire]	1.0	15 December 2017
Other [67_RETAKE Recruitment Poster - Family/Friend]	1.0	15 December 2017
Other [68_RETAKE Recruitment Poster - Ward Staff]	1.0	15 December 2017
Other [69_RETAKE Text Message Reminders for Questionnaires]	1.0	15 December 2017
Other [70_Participant Postal Questionnaire Cover Page]	1.0	15 December 2017
Other [71_Carer Postal Questionnaire Cover Page]	1.0	15 December 2017
Other [72_RETAKE Participant QTool Cover Page]	1.0	15 December 2017
Other [73_RETAKE Carer QTool Cover Page]	1.0	15 December 2017
Other [74_QTool Guidance Document]	1.0	15 December 2017
Other [75_QTool Login Details Email]	1.0	15 December 2017
Other [Applicant response to REC Staff pre validation requests]		10 January 2018
Other [45_RETAKE Cover Letter_CASE STUDIES_SS_v1.0_20171215]	1.0	15 December 2017
Participant consent form [13_RETAKE Participant Consent Form]	1.0	15 December 2017
Participant consent form [14_RETAKE Carer Consent Form]	1.0	15 December 2017
Participant consent form [15_RETAKE Carer Consent Form - Interviews]	1.0	15 December 2017
Participant consent form [16_RETAKE Employer Consent Form - Interviews]	1.0	15 December 2017
Participant consent form [17_RETAKE NHS Staff Consent Form - Interviews]	1.0	15 December 2017
Participant consent form [18_RETAKE Participant Consent Form - Interviews]	1.0	15 December 2017
Participant consent form [19_RETAKE Therapist Consent Form - Interviews]	1.0	15 December 2017
Participant consent form [20_RETAKE Participant Consent Form - Case Studies]	1.0	15 December 2017
Participant information sheet (PIS) [5_RETAKE Participant Info Sheet - SHORT VERSION]	1.0	15 December 2017
Participant information sheet (PIS) [7_RETAKE Carer Info Sheet - Interviews]	1.0	15 December 2017
Participant information sheet (PIS) [8_RETAKE Employer Info Sheet - Interviews]	1.0	15 December 2017
Participant information sheet (PIS) [9_RETAKE NHS Staff Info Sheet - Interviews]	1.0	15 December 2017
Participant information sheet (PIS) [10_RETAKE Participant Info Sheet - Interviews]	1.0	15 December 2017
Participant information sheet (PIS) [11_RETAKE Therapist Info Sheet - Interviews]	1.0	15 December 2017
Participant information sheet (PIS) [12_RETAKE Participant Info Sheet - Case Studies]	1.0	15 December 2017
Participant information sheet (PIS) [RETAKE Participant Info Sheet_v2.0_TRACKED CHANGES]	2.0	06 February 2018
Participant information sheet (PIS) [RETAKE Participant Info Sheet_v2.0_CLEAN]	2.0	06 February 2018

Participant information sheet (PIS) [RETAKE Carer Info Sheet_CLEAN]	2.0	06 February 2018
Research protocol or project proposal [2_RETAKE Protocol]	1.0	15 December 2017
Summary CV for Chief Investigator (CI) [3_CV - Kate Radford]		25 October 2017
Summary, synopsis or diagram (flowchart) of protocol in non technical language [81_RETAKE Study Summary]	1.0	15 December 2017
Validated questionnaire [52_Confidence after Stroke Measure]		
Validated questionnaire [53_Community Integration Questionnaire]		
Validated questionnaire [54_Caregiver Strain Index]		
Validated questionnaire [55_EQ-5D-5L]		
Validated questionnaire [56_Hospital Anxiety and Depression Scale]		
Validated questionnaire [57_Nottingham Extended Activities of daily Living Measure]		
Validated questionnaire [58_Oxford Cognitive Screen User Manual]		
Validated questionnaire [58_Oxford Cognitive Screen Sample Copy]		

You should ensure that the sponsor has a copy of the final documentation for the study. It is the sponsor's responsibility to ensure that the documentation is made available to R&D offices at all participating sites.

**18/EM/0019**	**Please quote this number on all correspondence**

Yours sincerely

Lindsey Wallace
REC Assistant

E-mail: NRESCommittee.EastMidlands-Nottingham2@nhs.net

Copy to: *Ms Angela Shone*
Shirley Mitchell, Nottinghamshire Healthcare NHS Foundation Trust
