# Supplementary material for: An individually randomised controlled multi-centre pragmatic trial with embedded economic and process evaluations of early vocational rehabilitation compared with usual care for stroke survivors: study protocol for the RETurn to work After stroKE (RETAKE) trial
Source: Trials. 2020 Dec 9;21:1010. doi: 10.1186/s13063-020-04883-1 (PMC7724443; doi:10.1186/s13063-020-04883-1)
Supplement: Supplementary file 9 — Additional file 9. [file 13063_2020_4883_MOESM9_ESM.pdf]

Dr Kathryn Radford
Associate Professor in Rehabilitation Research (Long Term Conditions)
University of Nottingham
Division of Rehabilitation and Ageing
B102, Medical School, Queen's Medical Centre (QMC)
Nottingham
NG7 2UH

Email: hra.approval@nhs.net

21 February 2018 – reissued to correct protocol assessment check

Dear Dr Radford

Letter of HRA Approval

**Study title:**	**RETurn to work After stroKE (RETAKE)Early vocational rehabilitation compared with usual care for stroke survivors: an individually randomised controlled multi-centre pragmatic trial with embedded economic and process evaluations**
**IRAS project ID:**	**234874**
**Protocol number:**	**17104**
**REC reference:**	**18/EM/0019**
**Sponsor**	**University of Nottingham**

I am pleased to confirm that **HRA Approval** has been given for the above referenced study, on the basis described in the application form, protocol, supporting documentation and any clarifications noted in this letter.

Participation of NHS Organisations in England

The sponsor should now provide a copy of this letter to all participating NHS organisations in England.

Appendix B provides important information for sponsors and participating NHS organisations in England for arranging and confirming capacity and capability. **Please read *Appendix B* carefully**, in particular the following sections:

- *Participating NHS organisations in England* – this clarifies the types of participating organisations in the study and whether or not all organisations will be undertaking the same activities
- *Confirmation of capacity and capability* - this confirms whether or not each type of participating NHS organisation in England is expected to give formal confirmation of capacity and capability. Where formal confirmation is not expected, the section also provides details on the time limit given to participating organisations to opt out of the study, or request additional time, before their participation is assumed.

- *Allocation of responsibilities and rights are agreed and documented (4.1 of HRA assessment criteria)* - this provides detail on the form of agreement to be used in the study to confirm capacity and capability, where applicable.

Further information on funding, HR processes, and compliance with HRA criteria and standards is also provided.

It is critical that you involve both the research management function (e.g. R&D office) supporting each organisation and the local research team (where there is one) in setting up your study. Contact details and further information about working with the research management function for each organisation can be accessed from the HRA website.

Appendices

The HRA Approval letter contains the following appendices:

- A – List of documents reviewed during HRA assessment
- B – Summary of HRA assessment

After HRA Approval

The document *"After Ethical Review – guidance for sponsors and investigators",* issued with your REC favourable opinion, gives detailed guidance on reporting expectations for studies, including:

- Registration of research
- Notifying amendments
- Notifying the end of the study

The HRA website also provides guidance on these topics, and is updated in the light of changes in reporting expectations or procedures.

In addition to the guidance in the above, please note the following:

- HRA Approval applies for the duration of your REC favourable opinion, unless otherwise notified in writing by the HRA.
- Substantial amendments should be submitted directly to the Research Ethics Committee, as detailed in the *After Ethical Review* document. Non-substantial amendments should be submitted for review by the HRA using the form provided on the HRA website, and emailed to hra.amendments@nhs.net.
- The HRA will categorise amendments (substantial and non-substantial) and issue confirmation of continued HRA Approval. Further details can be found on the HRA website.

Scope

HRA Approval provides an approval for research involving patients or staff in NHS organisations in England.

If your study involves NHS organisations in other countries in the UK, please contact the relevant national coordinating functions for support and advice. Further information can be found through IRAS.

If there are participating non-NHS organisations, local agreement should be obtained in accordance with the procedures of the local participating non-NHS organisation.

User Feedback

The Health Research Authority is continually striving to provide a high quality service to all applicants and sponsors. You are invited to give your view of the service you have received and the application procedure. If you wish to make your views known please use the feedback form available on the HRA website.

HRA Training

We are pleased to welcome researchers and research management staff at our training days – see details on the HRA website.

Your IRAS project ID is **234874**. Please quote this on all correspondence.

Yours sincerely

Michael Pate
Assessor

Email: hra.approval@nhs.net

Copy to: *Ms Angela Shone – University of Nottingham – Sponsor contact*
Ms Shirley Mitchell - Nottinghamshire Healthcare NHS Foundation Trust – Lead NHS R&D contact
Ms Vicki McLellan – University of Leeds - Trial Manager.

Appendix A - List of Documents

The final document set assessed and approved by HRA Approval is listed below.

Document	*Version*	*Date*
Contract/Study Agreement template [82_Model non-commercial Agreement]		
Contract/Study Agreement template		
Contract/Study Agreement template [82_Model non-commercial Agreement]		
Covering letter on headed paper	1.0	15 December 2017
Covering letter on headed paper [01_RETAKE REC Cover Letter]	1.0	15 December 2017
Covering letter on headed paper [01_RETAKE REC Cover Letter]	1.0	06 February 2018
Evidence of Sponsor insurance or indemnity (non NHS Sponsors only) [83_Sponsor Insurance]		
GP/consultant information sheets or letters [46_RETAKE GP Letter]	1.0	15 December 2017
HRA Schedule of Events	1	11 January 2018
HRA Statement of Activities	1	11 January 2018
Interview schedules or topic guides for participants [47_RETAKE Carer Topic Guide - Interviews]	1.0	15 December 2017
Interview schedules or topic guides for participants [48_RETAKE Employer Topic Guide - Interviews]	1.0	15 December 2017
Interview schedules or topic guides for participants [49_RETAKE NHS Staff Topic Guide - Interviews]	1.0	15 December 2017
Interview schedules or topic guides for participants [50_RETAKE Participant Topic Guide - Interviews]	1.0	15 December 2017
Interview schedules or topic guides for participants [51_RETAKE Therapist Topic Guide - Interviews]	1.0	15 December 2017
IRAS Application Form [IRAS_Form_20122017]		20 December 2017
IRAS Checklist XML [Checklist_04012018]		04 January 2018
Letter from funder [76_Funder Letter]		
Letter from sponsor [84_Sponsor Letter]		
Letters of invitation to participant [22_RETAKE Postal Consent to Follow-up Leaflet - Stroke Survivor]	2.0	06 February 2018
Letters of invitation to participant [23_RETAKE Consent to Follow-up Leaflet - Stroke Survivor]	2.0	06 February 2018
Letters of invitation to participant [21_RETAKE Postal Approach Cover Letter - Stroke Survivor]	1.0	15 December 2017
Letters of invitation to participant [22_RETAKE Postal Consent to Follow-up Leaflet - Stroke Survivor]	1.0	15 December 2017
Letters of invitation to participant [23_RETAKE Consent to Follow-up Leaflet - Stroke Survivor]	1.0	15 December 2017
Letters of invitation to participant [24_RETAKE Allocation Letter]	1.0	15 December 2017
Letters of invitation to participant [27_RETAKE Participant QTool Follow-up Email]	1.0	15 December 2017
Letters of invitation to participant [33_RETAKE Postal Consent to Follow-up Leaflet - Carer]	1.0	15 December 2017
Letters of invitation to participant [37_RETAKE Carer QTool Follow-up Email]	1.0	15 December 2017
Letters of invitation to participant [38_RETAKE Carer QTool Follow-up Reminder Email]	1.0	15 December 2017

Letters of invitation to participant [41_RETAKE Carer Cover Letter - Interviews]	1.0	15 December 2017
Letters of invitation to participant [43_RETAKE NHS Staff Cover Letter - Interviews]	1.0	15 December 2017
Letters of invitation to participant [45_RETAKE Participant Cover Letter - Interviews]	1.0	15 December 2017
Letters of invitation to participant [42_RETAKE Employer Cover Letter - Interviews]	1.0	15 December 2017
Letters of invitation to participant [39_RETAKE Carer Thank You Letter (3/6m)]	1.0	15 December 2017
Letters of invitation to participant [40_RETAKE Carer Thank You Letter (12m)]	1.0	15 December 2017
Letters of invitation to participant [34_RETAKE Consent to Follow-up Leaflet - Carer]	1.0	15 December 2017
Letters of invitation to participant [35_RETAKE Carer Follow-up Letter]	1.0	15 December 2017
Letters of invitation to participant [36_RETAKE Carer Follow-up Reminder Letter]	1.0	15 December 2017
Letters of invitation to participant [28_RETAKE Participant QTool Follow-up Reminder Email]	1.0	15 December 2017
Letters of invitation to participant [29_RETAKE Participant Thank You Letter (3/6m)]	1.0	15 December 2017
Letters of invitation to participant [30_RETAKE Participant Thank You Letter (12m)]	1.0	15 December 2017
Letters of invitation to participant [31_RETAKE QTool Thank You Message]	1.0	15 December 2017
Letters of invitation to participant [32_RETAKE Carer Postal Approach Cover Letter]	1.0	15 December 2017
Letters of invitation to participant [RETAKE Participant Follow-up Letter]	1.0	15 December 2017
Letters of invitation to participant [26_RETAKE Participant Follow-up Reminder Letter]	1.0	15 December 2017
Letters of invitation to participant [44_RETAKE Participant Cover Letter - Interviews]	1.0	15 December 2017
Non-validated questionnaire [60_RETAKE 3m Participant Health Economics Questionnaire]	1.0	15 December 2017
Non-validated questionnaire [61_RETAKE 6m Participant Health Economics Questionnaire]	1.0	15 December 2017
Non-validated questionnaire [62_RETAKE 12m Participant Health Economics Questionnaire]	1.0	15 December 2017
Non-validated questionnaire [63_RETAKE Baseline Carer Health Economics Questionnaire]	1.0	15 December 2017
Non-validated questionnaire [64_RETAKE 3m Carer Health Economics Questionnaire]	1.0	15 December 2017
Non-validated questionnaire [65_RETAKE 6m Carer Health Economics Questionnaire]	1.0	15 December 2017
Non-validated questionnaire [66_RETAKE 12m Carer Health Economics Questionnaire]	1.0	15 December 2017
Non-validated questionnaire [59_RETAKE Baseline Participant Health Economics Questionnaire]	1.0	15 December 2017
Other [67_RETAKE Recruitment Poster - Family/Friend]	1.0	15 December 2017
Other [68_RETAKE Recruitment Poster - Ward Staff]	1.0	15 December 2017
Other [69_RETAKE Text Message Reminders for Questionnaires]	1.0	15 December 2017
Other [70_Participant Postal Questionnaire Cover Page]	1.0	15 December 2017
Other [71_Carer Postal Questionnaire Cover Page]	1.0	15 December 2017

Other [72_RETAKE Participant QTool Cover Page]	1.0	15 December 2017
Other [73_RETAKE Carer QTool Cover Page]	1.0	15 December 2017
Other [74_QTool Guidance Document]	1.0	15 December 2017
Other [75_QTool Login Details Email]	1.0	15 December 2017
Other [Applicant response to REC Staff pre validation requests]		10 January 2018
Other [45_RETAKE Cover Letter_CASE STUDIES_SS_v1.0_20171215]	1.0	15 December 2017
Other [Details of sites being approached following initial assessment]		25 January 2018
Participant consent form [13_RETAKE Participant Consent Form]	1.0	15 December 2017
Participant consent form [14_RETAKE Carer Consent Form]	1.0	15 December 2017
Participant consent form [15_RETAKE Carer Consent Form - Interviews]	1.0	15 December 2017
Participant consent form [16_RETAKE Employer Consent Form - Interviews]	1.0	15 December 2017
Participant consent form [17_RETAKE NHS Staff Consent Form - Interviews]	1.0	15 December 2017
Participant consent form [18_RETAKE Participant Consent Form - Interviews]	1.0	15 December 2017
Participant consent form [19_RETAKE Therapist Consent Form - Interviews]	1.0	15 December 2017
Participant consent form [20_RETAKE Participant Consent Form - Case Studies]	1.0	15 December 2017
Participant information sheet (PIS) [4_RETAKE Participant Info Sheet]	1.0	15 December 2017
Participant information sheet (PIS) [5_RETAKE Participant Info Sheet - SHORT VERSION]	1.0	15 December 2017
Participant information sheet (PIS) [6_RETAKE Carer Info Sheet]	1.0	15 December 2017
Participant information sheet (PIS) [7_RETAKE Carer Info Sheet - Interviews]	1.0	15 December 2017
Participant information sheet (PIS) [8_RETAKE Employer Info Sheet - Interviews]	1.0	15 December 2017
Participant information sheet (PIS) [9_RETAKE NHS Staff Info Sheet - Interviews]	1.0	15 December 2017
Participant information sheet (PIS) [10_RETAKE Participant Info Sheet - Interviews]	1.0	15 December 2017
Participant information sheet (PIS) [11_RETAKE Therapist Info Sheet - Interviews]	1.0	15 December 2017
Participant information sheet (PIS) [12_RETAKE Participant Info Sheet - Case Studies]	1.0	15 December 2017
Participant information sheet (PIS) [RETAKE Participant Info Sheet_v2.0_TRACKED CHANGES]	2.0	06 February 2018
Participant information sheet (PIS) [RETAKE Participant Info Sheet_v2.0_CLEAN]	2.0	06 February 2018
Participant information sheet (PIS) [RETAKE Carer Info Sheet_CLEAN]	2.0	06 February 2018
Research protocol or project proposal [2_RETAKE Protocol]	1.0	15 December 2017
Summary CV for Chief Investigator (CI) [3_CV - Kate Radford]		25 October 2017
Summary, synopsis or diagram (flowchart) of protocol in non technical language [81_RETAKE Study Summary]	1.0	15 December 2017
Validated questionnaire [52_Confidence after Stroke Measure]		
Validated questionnaire [53_Community Integration Questionnaire]		
Validated questionnaire [54_Caregiver Strain Index]		

Validated questionnaire [55_EQ-5D-5L]		
Validated questionnaire [56_Hospital Anxiety and Depression Scale]		
Validated questionnaire [57_Nottingham Extended Activities of daily Living Measure]		
Validated questionnaire [58_Oxford Cognitive Screen User Manual]		
Validated questionnaire [58_Oxford Cognitive Screen Sample Copy]		

Appendix B - Summary of HRA Assessment

This appendix provides assurance to you, the sponsor and the NHS in England that the study, as reviewed for HRA Approval, is compliant with relevant standards. It also provides information and clarification, where appropriate, to participating NHS organisations in England to assist in assessing and arranging capacity and capability.

For information on how the sponsor should be working with participating NHS organisations in England, please refer to the, *participating NHS organisations, capacity and capability* and *Allocation of responsibilities and rights are agreed and documented (4.1 of HRA assessment criteria)* sections in this appendix.

The following person is the sponsor contact for the purpose of addressing participating organisation questions relating to the study:

Name: Ms Angela Shone

Email: sponsor@nottingham.ac.uk

HRA assessment criteria

Section	HRA Assessment Criteria	Compliant with Standards	Comments
1.1	IRAS application completed correctly	Yes	Stroke services at NHS Trust sites across the UK will be approached to determine if they wish to take part in the trial. Sites expressing an interest will be invited to complete a questionnaire to determine that appropriate services are available to support the trial. A summary of participating centres, including screening and reasons for non-selection, will be maintained by CTRU.<br><br>In addition to the sites listed in part C of the IRAS form, the following sites will be participating:<br><br>• Norfolk Community Health and Care Trust<br><br>• East Lancashire Hospitals NHS Trust<br><br>• South West Yorkshire Partnership NHS Foundation trust

Section	HRA Assessment Criteria	Compliant with Standards	Comments
			• Rotherham Doncaster and South Humber NHS Foundation Trust<br><br>• Guy's and St Thomas NHS Foundation Trust<br><br>• Hounslow and Richmond Community Healthcare NHS Trust<br><br>• Royal Devon and Exeter NHS Foundation Trust<br><br>• Bridgewater Community Healthcare NHS Foundation Trust<br><br>• South Tees Hospitals NHS Foundation Trust
2.1	Participant information/consent documents and consent process	Yes	Patients will be approached by post to take part in interviews at the end of the study – They will be provided with an information sheet and then contacted by phone to check interest. If the participant is interested a suitable time to conduct the interview will be agreed and consent will be sought prior to this taking place.<br><br>Carers will be approached by post to take part in interviews at the end of the study – They will be provided with an information sheet and then contacted by phone to check interest. If the participant is interested a suitable time to conduct the interview will be agreed and consent will be sought prior to this taking place.<br><br>The short version of the PIS will be provided to potential participants who are approached face to face as an introduction to the study – If they are interested in participating they will be provided with the standard information sheet ahead of consent being taken.

Section	HRA Assessment Criteria	Compliant with Standards	Comments
			Intervention therapists will be identified through discussions with the relevant clinical teams at each site during site set-up – the research team will be guided by the clinical team at site to identify willing and appropriate OTs to be trained in and deliver the intervention.<br><br>NHS staff will be identified by discussion with the local lead at each site. The covering letter will be sent by the PI and consent will be taken by the Research Assistant if the staff member is interested in taking part.<br><br>Recruitment posters will be displayed in public areas and wards at each participating site to allow family members/friends to contact the research team for more information about the RETAKE study. No identifiable/contact details would be provided to the research team, once the relative/friend has spoken with the research team they may then speak to the potential participant who may then contact the research team directly if they wish.
3.1	Protocol assessment	Yes	ONS data will not now be collected for this study, and the protocol will be amended as part of the next amendment.
4.1	Allocation of responsibilities and rights are agreed and documented	Yes	A modified mNCA will form the agreement between the sponsor and sites. Justifications for the modifications are provided in the enclosed Statement of Activities.

Section	HRA Assessment Criteria	Compliant with Standards	Comments
4.2	Insurance/indemnity arrangements assessed	Yes	The University of Nottingham insurance indemnifies the University staff for their role in the research, including the interviews.<br><br>Where applicable, independent contractors (e.g. General Practitioners) should ensure that the professional indemnity provided by their medical defence organisation covers the activities expected of them for this research study
4.3	Financial arrangements assessed	Yes	Funded by NIHR HTA grant. Funding to sites is detailed in the Statement of Activities provided.
5.1	Compliance with the Data Protection Act and data security issues assessed	Yes	Consent to follow-up forms that are completed whilst the patient is still in hospital will be handed to ward staff who will store the forms securely on the ward before being collected by the research team. Potential participants who are approached by post and provided with a consent to follow-up leaflet will also be provided with a pre-paid envelope addressed to the research team to allow return of this form via standard Royal Mail.<br><br>In addition to trial number, the CTRU routinely collects participant initials, DoB and NHS number on consent forms which are sent from the treating institution to the CTRU. The reason we include these identifiers is two-fold. Firstly, whilst trial ID number provides a unique identifier, it can be misread or incorrectly transcribed during CRF completion. Collection of initials, DOB and NHS number provides an essential contingency measure which ensures CTRU staff can correctly identify mis-transcribed trial identifiers and therefore avoid recording data against the wrong

Section	HRA Assessment Criteria	Compliant with Standards	Comments
			patient. Secondly, whilst trial ID numbers are essential in the efficient management of trial patient records within the CTRU, they carry very little meaning to clinical and healthcare staff working in the treating institution who are used to routinely using patient names in tandem with DOB and NHS number when managing patients. The combination of just trial ID and DOB, or just trial ID and initials, would not provide a satisfactory means of tracking patients and efficiently managing data.<br><br>Trial data on paper will be sent from sites to the CTRU via standard Royal Mail (and in some cases by secure email). All data recorded on paper forms will be anonymised and any personal/identifiable data will be sent and stored at the CTRU separately to other trial data. Clear guidance will be provided to all sites re transfer and storage of trial data.<br><br>Personal data will not be collected as part of the questionnaires. Trial ID, initials and DoB will be collected as a means of identifying participants.<br><br>All personal data will be stored at the CTRU in locked cabinets separately from the main trial data – only authorised members of the study team will have access to this data for the purposes of follow-up. This data will be stored for 6 – 12 months after the study has ended before being destroyed.<br><br>The information sheet was updated in response to the REC provisional opinion to state that GP will be contacted for the participant's contact details prior to sending questionnaires.

Section	HRA Assessment Criteria	Compliant with Standards	Comments
			Consent forms will be sent from sites to the CTRU via standard Royal Mail (and in some cases by secure email) separately to other trial data. Consent forms will be stored at the CTRU in locked cabinets separately from the main trial data.<br><br>A Research Assistant based at King's College London will be conducting and audio recording the interviews. A 3rd party transcriber (who KCL have employed previously) will complete the transcriptions.<br><br>At the end of the study, data, including interview data and transcripts, will be securely stored at the University of Nottingham for a minimum of 7 years and then destroyed. Only relevant members of the study team at the University of Nottingham will have access to this data during this time.<br><br>Any data transferred from the CTRU to other collaborators for the purposes of analysis will be sent via the CTRU's secure file transfer system which uses SSL encryption to transfer all messages and attachments. All users of this system (both senders and recipients) must have a unique log in to access to the system.
5.2	CTIMPS – Arrangements for compliance with the Clinical Trials Regulations assessed	Not Applicable	No comments
5.3	Compliance with any applicable laws or regulations	Yes	No comments
6.1	NHS Research Ethics Committee favourable opinion received for applicable studies	Yes	No comments

Section	HRA Assessment Criteria	Compliant with Standards	Comments
6.2	CTIMPS – Clinical Trials Authorisation (CTA) letter received	Not Applicable	No comments
6.3	Devices – MHRA notice of no objection received	Not Applicable	No comments
6.4	Other regulatory approvals and authorisations received	Not Applicable	No comments

Participating NHS Organisations in England

This provides detail on the types of participating NHS organisations in the study and a statement as to whether the activities at all organisations are the same or different.

All participating organisations will be conducting the same activities, as per the protocol. Therefore, one site type.

The Chief Investigator or sponsor should share relevant study documents with participating NHS organisations in England in order to put arrangements in place to deliver the study. The documents should be sent to both the local study team, where applicable, and the office providing the research management function at the participating organisation. For NIHR CRN Portfolio studies, the Local LCRN contact should also be copied into this correspondence. For further guidance on working with participating NHS organisations please see the HRA website.

If chief investigators, sponsors or principal investigators are asked to complete site level forms for participating NHS organisations in England which are not provided in IRAS or on the HRA website, the chief investigator, sponsor or principal investigator should notify the HRA immediately at hra.approval@nhs.net. The HRA will work with these organisations to achieve a consistent approach to information provision.

Confirmation of Capacity and Capability

This describes whether formal confirmation of capacity and capability is expected from participating NHS organisations in England.
Participating NHS organisations in England **will be expected to formally confirm their capacity and capability to host this research**.<br>• Following issue of this letter, participating NHS organisations in England may now confirm to the sponsor their capacity and capability to host this research, when ready to do so. How capacity and capacity will be confirmed is detailed in the *Allocation of responsibilities and rights are agreed and documented (4.1 of HRA assessment criteria)* section of this appendix.<br>• The Assessing, Arranging, and Confirming document on the HRA website provides further information for the sponsor and NHS organisations on assessing, arranging and confirming capacity and capability.

Principal Investigator Suitability

This confirms whether the sponsor position on whether a PI, LC or neither should be in place is correct for each type of participating NHS organisation in England and the minimum expectations for education, training and experience that PIs should meet (where applicable).
A local Principal Investigator should be in place at each participating site.<br><br>The training to be offered by the Sponsor and what the Sponsor expects to have been completed is clear in the Statement of Activities provided.<br><br>GCP training is not a generic training expectation, in line with the HRA/MHRA statement on training expectations.

HR Good Practice Resource Pack Expectations

This confirms the HR Good Practice Resource Pack expectations for the study and the pre-engagement checks that should and should not be undertaken
Screening, consent and the baseline assessment will be conducted by local staff holding a contract with the participating site. This is also true of the intervention and the activities taking place as part of usual care.<br><br>For interviews conducted over the telephone with NHS patients or carers, a Letter of Access would be required for researchers not already having a contract with a participating site. No evidence of pre-engagement check would be required. This is the same for NHS staff interviews where those interviews take place within a location where NHS care is provided.<br><br>For face-to-face interviews with patients or carers, a Letter of Access would be required for researchers not already holding a contract with the participating site. Evidence of standard DBS and OH clearance would be expected.

Other Information to Aid Study Set-up

This details any other information that may be helpful to sponsors and participating NHS organisations in England to aid study set-up.
• The applicant has indicated that they intend to apply for inclusion on the NIHR CRN Portfolio.<br>• The copyright associated with the anticipated amended manual and training programme will be managed through the University of Nottingham Technology Transfer Office with liaison with relevant parties such as Nottingham University hospitals NHS Trust and Nottingham Citycare partnership. The IP will be disseminated alongside the research findings to a widespread audience including participants, commission groups, community-based organisations, policy makers, media, Nottingham University and other universities. Oral/poster presentations and workshops at sponsor hosted events, community meetings and national and international scientific conferences will be one method of recognition and dissemination. Leverage of existing resources, relationships, and networks will be utilised fully including involvement from knowledge transfer professionals/materials. If the trial is positive we will make an application to the Academic Health Science Network to roll the technology out across clinical services (e.g. Community stroke rehabilitation teams, occupational therapy departments) using appropriate training workshops, in a way that will ensure both utilisers and referring agencies fully understand the scope of the manual. The manual would then be created in mass to enable both national and eventual international distribution.
